# SpaConTDS: A multimodal contrastive learning framework for identifying spatial domains by applying tuple disturbing strategy

**DOI:** 10.1371/journal.pcbi.1013893

**Published:** 2026-01-29

**Authors:** Ruiwen Xu, Xiaoqing Cheng, Waiki Ching, Siyao Wu, Yuanben Zhang, Yidan Zhang

**Affiliations:** 1 School of Mathematics and Statistics, Xi’an Jiaotong University, Shaanxi, China; 2 Department of Mathematics, The University of Hong Kong, Hong Kong, China; 3 Sina Weibo Beijing, China; 4 Aerospace Information Research Institute, Chinese Academy of Science, Beijing, China; University of Pittsburgh, UNITED STATES OF AMERICA

## Abstract

The rational utilization of multimodal spatial transcriptomics (ST) data enables accurate identification of spatial domains, which is essential for investigating cellular structure and functions. In this study, we proposed SpaConTDS, a novel framework that integrates reinforcement learning with self-supervised multimodal contrastive learning. SpaConTDS generates positive and negative samples through data augmentation and a pseudo-label tuple perturbation strategy, enabling the learning of fused representations that capture global semantics and cross-modal interactions. The model’s hyper-parameters are dynamically optimized using reinforcement learning. Extensive experiments across various resolutions and platforms demonstrate that SpaConTDS achieves state-of-the-art accuracy in spatial domain identification and outperforms existing methods in downstream tasks such as denoising, trajectory inference, and UMAP visualization. Moreover, SpaConTDS effectively integrates multiple tissue sections and corrects batch effects without requiring prior alignment. Compared to existing approaches, SpaConTDS offers more robust fused representations of multimodal data, providing researchers with a flexible and powerful tool for a wide range of spatial transcriptomics analyses.

## Author summary

Advancements in ST technologies have enabled researchers to simultaneously capture histological features, gene expression profiles, and spatial information. Unsupervised clustering of captured spots into spatial domains constitutes a fundamental component of spatial transcriptomics analysis, with the objective of delineating spatially coherent regions that are typically associated with distinct biological functions or tissue architectures. In this paper, we proposed a multi-modal spatial domain identification model called SpaConTDS. The innovations of SpaConTDS can be summarized as follows. Firstly, it utilizes a self-supervised multimodal contrastive learning method to effectively integrate gene expression and histopathological image information for spatial domain identification and alignment-free slice integration. Secondly, SpaConTDS uses reinforcement learning and global positive/negative sample construction strategies to adaptively capture fused representations that encompass interactions between modalities, which ensures that weak modalities are not neglected while avoiding the introduction of noise from image information. Thirdly, for multi-slice integration, negative samples and positive samples derived from the global similarity matrix can cover all slices, enabling SpaConTDS to automatically smooth the features of adjacent points both within and across slices without the need for slice alignment, thus learning more comprehensive cross-slice information and alleviating batch effects. The numerical results demonstrate that SpaConTDS exhibits superiority over existing methods in both spatial domain identification and integrated analysis on multiple slices. Moreover, the learned representations are applicable to various downstream tasks, including trajectory inference, gene expression denoising and uniform manifold approximation and projection (UMAP) visualization.

## Introduction

Spatial transcriptomics (ST) is an innovative molecular profiling technology that enables the measurement of gene expression at distinct tissue locations and the capture of its spatial distribution within tissue slices [1]. The spatially resolved information allows researchers to gain deeper insights into cellular functions and the underlying mechanisms of disease pathology. Advancements in ST technologies have enabled researchers to simultaneously capture histological features, gene expression profiles, and spatial information that is not accessible through single-cell RNA sequencing (scRNA-seq) alone [2]. Spatial information is crucial for analyzing the impact of the microenvironment on cellular functions, uncovering the biological characteristics and pathological mechanisms of diseases [3], and facilitating the inference of intercellular communication, particularly juxtacrine signaling [4]. Popularly used spatial resolved transcriptomics platforms include in situ hybridization (ISH) technologies (e.g., seqFISH [5], seqFISH+ [6], MERFISH [7]), sequencing-based technologies (ISS) (e.g., STARmap [8], 10x Xenium [9]), and in situ capture (ISC) technologies (e.g., HDST [10], ST [11], Slide-seq [12], 10x Visium [13]).

Unsupervised clustering of captured spots into spatial domains constitutes a fundamental component of spatial transcriptomics analysis, with the objective of delineating spatially coherent regions that are typically associated with distinct biological functions or tissue architectures. The identified spatial domains are expected to exhibit spatial continuity, reflecting the inherent organization of tissue architecture and ensuring biological interpretability of the clustering results. However, traditional clustering algorithms, such as k-means [14], Louvain [15], and Leiden [15], rely exclusively on gene expression data, without incorporating spatial context, which often leads to the identification of spatial domains that are fragmented or lack spatial continuity. Several clustering methods have been specially developed for ST analysis, with certain approaches improving spatial domain identification by incorporating spatial proximity or similarity between neighboring spots. For example, BayesSpace [16] employs a fully Bayesian framework to integrate spatial correlation by incorporating spatial neighborhood structure into the prior information. STAGATE [17] utilizes an attention-based convolutional networks to reconstruct gene expression profiles, enabling the adaptive learning of spatial and gene expression data. ConST [18] adopts a contrastive learning framework comprising three hierarchical tasks to learn a representative low-dimensional embedding that is robust to noise perturbations and preserves cluster-level similarity. GraphST [19], on the other hand, integrates graph neural networks with self-supervised contrastive learning to enhance information extraction. In contrast, ConSpaS [20] combines both global and local similarities to identify spatial domains by integrating graph autoencoders with augmentation-free contrastive learning. Several methods leverage single-cell RNA sequencing data to enhance spatial domain identification. For example, IRIS [21] uses single-cell RNA sequencing as a reference to inform spatial domain detection in SRT studies, integrating multiple slices while modeling intra- and inter-slice correlations to achieve high accuracy and efficiency. However, spatial transcriptomics data often lack matched single-cell RNA datasets in practice, limiting the broad applicability of such approaches and underscoring the need for more flexible integration strategies.

On the other hand, in medical field, spatial transcriptomics sequencing is often accompanied by the preservation of histological imaging data to provide complementary spatial context for gene expression analysis. And those tissue pathology images, such as hematoxylin and eosin (H&E) stained images, have been demonstrated to contain morphological features, which can be used to predict gene expressioncitations. Consequently, clustering methods that disregard histological context may yield spatial domains with ambiguous boundaries and inconsistencies when compared to expert annotations. To fully leverage the rich information embedded in histology images, several computational methods have been developed to integrate image features with spatial transcriptomic data. For instance, stLearn [22] performs gene expression normalization prior to clustering by incorporating spatial coordinates and morphological features extracted from histology images to calculate inter-spot distances. ConGI [23] adopts a multimodal contrastive learning to learn a joint representation of gene expression and histology images at the spot level for spatial domain identification. Although ConGI treats image features as a separate modality during training, it simply applies a simple linear fusion of the two modalities and does not fully exploit the interactions between them. Furthermore, fixed weighting schemes typically involve manual parameter selection, which may introduce variability across applications. Both of the aforementioned methods extract features from individual modalities prior to normalization or feature fusion. However, this sequential processing strategy may result in the loss of critical information or the introduction of modality-specific noise, thereby limiting its overall performance. Empirically, strategies that prioritize multimodal integration before representation learning have demonstrated improved accuracy and robustness. For instance, SpaGCN [24] integrates gene expression, spatial coordinates and color features derived from histology images using graph convolutional networks (GCN) [25] to identify spatial domains. However, SpaGCN leverages only the color features of histology images, without fully exploring the broader spectrum of morphological information. Moreover, both the complexity of multimodal information integration and the risk of introducing redundant or irrelevant features must be carefully considered. Several multimodal spatial domain identification methods integrate multi-omics data, such as MISO [26] and MorphLink [27]. MISO integrates multiple spatial omics modalities by learning modality-specific embeddings and their interaction features via outer products. MorphLink introduces a curve-based pattern similarity index (CPSI) to associate tissue morphology with molecular dynamics by quantifying local and global spatial pattern similarities in spatial omics data.

The issue of balancing strong and weak modalities is a non-trivial challenge in multi-modal data analysis, yet it remains largely unaddressed in the aforementioned models. This imbalance may lead to dominant modalities overshadowing weaker ones during fusing process, thereby reducing the overall effectiveness and robustness of the model. Therefore, selecting appropriate multimodal integration strategies according to spatial transcriptomics data’s characteristics, while ensuring a balanced contribution from all modalities, is of critical importance for achieving optimal performance. Furthermore, spatial transcriptomics technologies are inherently constrained by the limited area that can be captured during a single acquisition. To study the entire tissue region of interest, samples are typically sectioned vertically or horizontally into multiple adjacent or consecutive slices. As a result, ST data sets often comprise several sections derived from the same tissue specimen. To fully leverage cross-slice information, these sections must be jointly analyzed, which is essential for constructing comprehensive representations of spatial organization and can substantially enhance the accuracy of spatial domain identification. Although several methods have been proposed for aligning ST data, they typically require manual registration, incur high computational costs, and often rely on coordinate-based alignment across slices. Prior to spatial domain identification, aligning slices using ST data alignment algorithms often entails substantial computational overhead and is highly dependent on the performance of the alignment models. Moreover, most existing methods do not incorporate histology image information, and consequently fail to capture subtle morphological and structural differences across tissue sections. These limitations undermine the accuracy and robustness of multi-slice spatial domain identification.

To overcome the above limitations, we developed SpaConTDS, which utilizes a self-supervised multimodal contrastive learning method to effectively integrate gene expression and histopathological image information for spatial domain identification and alignment-free slice integration. SpaConTDS uses reinforcement learning and global positive/negative sample construction strategies to adaptively capture fused representations that encompass interactions between modalities, which ensures that weak modalities are not neglected while avoiding the introduction of noise from image information. For multi-slice integration, negative samples and positive samples derived from the global similarity matrix can cover all slices, enabling SpaConTDS to automatically smooth the features of adjacent points both within and across slices without the need for slice alignment, thus learning more comprehensive cross-slice information and alleviating batch effects. We extensively tested SpaConTDS on various ST datasets generated from different platforms. The results demonstrate that SpaConTDS exhibits superiority over existing methods in both spatial domain identification and integrated analysis on multiple slices. Moreover, the learned representations are applicable to various downstream tasks, including trajectory inference, gene expression denoising and uniform manifold approximation and projection (UMAP) visualization.

## Results

### SpaConTDS identifies morphological structures of heterogeneous tissues

**Fig 1:**
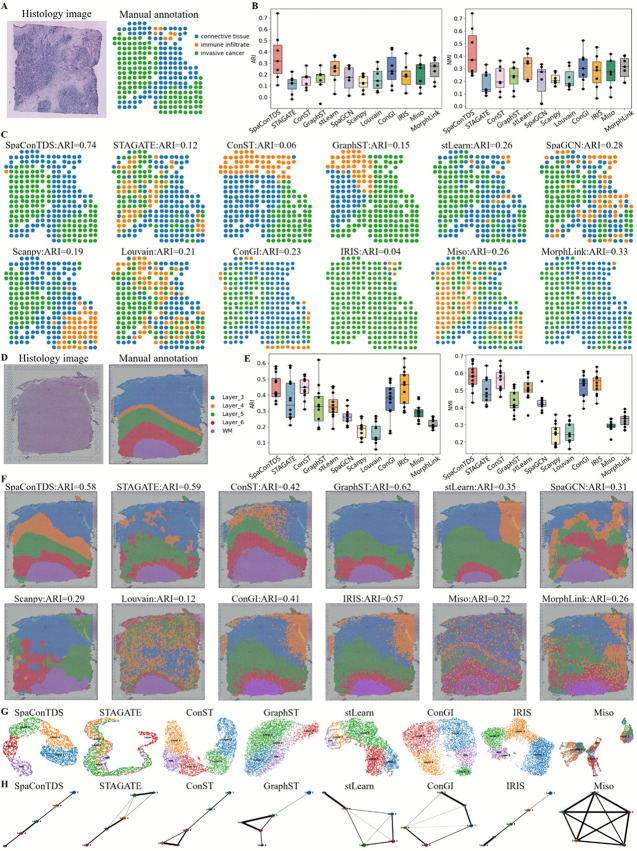
SpaConTDS facilitates accurate spatial domain identification and downstream tasks in HER2+ and DLPFC. (**A**) H&E stained image and manual annotations of HER2+ section D1. (**B**) Boxplots of ARI and NMI of the twelve methods applied to all 7 HER2+ sections. (**C**) Clustering results with ARI of SpaConTDS and baseline methods on HER2+ section D1. (**D**) H&E stained image and manual annotations of DLPFC slice 151671. (**E**) Boxplots of ARI and NMI of the twelve methods applied to all 12 DLPFC slices. (**F**) Clustering results with ARI of SpaConTDS and baseline methods on DLPFC slice 151671. (**G**) UMAP visualization of SpaConTDS and baseline methods on DLPFC slice 151671. (**H**) PAGA trajectory graphs of SpaConTDS and baseline methods on DLPFC slice 151671.

We first evaluated the spatial clustering performance of SpaConTDS on the HER2-positive breast tumor (HER2+) dataset generated using the ST platform, which comprises seven sections, each containing 3-6 manually annotated spatial domains [28]. We compared SpaConTDS with a non-spatial clustering method (Louvain) and 10 state-of-the-art spatial clustering methods, including ConST, STAGATE, GraphST, scanpy, SpaGCN, stLearn, ConGI, IRIS, MISO and MorphLink, model details and parameter settings are provided in S1 Text Sect 2. The evaluation focused on each method’s ability to recover the annotated anatomical cortex layers in an unsupervised manner. Across all seven sections, SpaConTDS achieved the highest median adjusted Rand index (ARI) and the highest median normalized mutual information(NMI), with only stLearn showing comparable performance, substantially outperforming other baseline methods (Fig 1B and 1C). Notably, in section D1, SpaConTDS achieved the highest ARI of 0.74, demonstrating spatial domain delineations that aligned more closely with manual annotations, and significantly surpassed the performance of other methods (Fig 1A and 1C).

Interestingly, we observed that the median ARI values for all methods were below 0.5, and the best-performing method varied across different sections. Although SpaConTDS achieved the best overall performance, its results in sections A1 and G2 (S1-S2 Figs) was inferior to other methods (A1: SpaConTDS, ARI = 0.11; IRIS, ARI = 0.39; G2: SpaConTDS, ARI = 0.18; MISO, ARI = 0.29). This performance degradation may be attributed to the low number of spots and the high proportion of missing data. The HER2+ dataset contained fewer than 700 spots per section, with an average missing rate of 85.3%. This extreme sparsity likely limited the modality’s ability to capture sufficient information during training, thereby hindering the extraction of effective feature representations and impairing its capacity to learn the global structure of the data.

Therefore, we further applied SpaConTDS to the human dorsolateral prefrontal cortex (DLPFC) dataset in 10x Visium, which comprises spatially resolved transcriptomic profiles of 12 slices [29], and each slice depictes four or six layers of the human dorsolateral prefrontal cortex and white matter (WM). Across all 12 slices, SpaConTDS achieved the third high median ARI (Figs 1E, S3, and S4). While alternative methods (e.g., ConST, IRIS) exhibited variable performance, with sporadic instances of outperforming SpaConTDS, our method consistently maintained stabilty, as evidenced by its narrower interquartile range in comparative boxplot analysis. This suggests that SpaConTDS is less susceptible to slice-specific variability, reinforcing its reliability for spatially heterogeneous datasets.

Notably, SpaConTDS achieved an medium Normalized Mutual Information (NMI) of 0.58, significantly out-performing all competing methods (Fig 1E), with IRIS(NMI = 0.57) being the second best. This enhanced performance likely stems from two key factors: (1) SpaConTDS generates cluster assignments that maintain high mutual information with ground truth annotations, and (2) its robustness to label permutation artifacts, as NMI is invariant to label ordering.

Specifically in slice 151671, containing 4110 spots and 19,020 genes, SpaConTDS demonstrated the most distinct and continuous cortical boundaries (ARI = 0.58, third-highest), accurately capturing the spatial relationship between WM layer andcortical layers (Fig 1D and 1E). Although GraphST achieved a marginally higher ARI (0.62), it exhibited a complete misidentification of layer 4, and it’s a limitation shared by other benchmarked methods to varying degrees; and the hierarchical structure obtained by the STAGATE method is not sufficiently clear (Fig 1F). Notably, the non-spatial Louvain algorithm performed poorest (ARI = 0.12), producing fragmented domain boundaries, indicating the necessity of spatial information in spatial domain identification problem. SpaConTDS, in contrast, was the only method to correctly reconstruct layer 4 with high morphological fidelity to manual annotations, further validating its precision in identifying tissue structures.

To further validate the biological insights revealed by SpaConTDS, we performed Uniform Manifold Approximation and Projection (UMAP) visualization on this slice, followed by pseudo-trajectory inference analysis. As shown in Fig 1G, while competing methods exhibited varying degrees of inter-layer spot mixing in there UMAP projections, SpaConTDS achieved a clearer inter-cluster boundaries. Its PAGA trajectory from Layer 3 to the white matter (WM) displayed an ideal linear progression (Fig 1H), demonstrating an almost linear developmental relationship across cortical Layer 3 to Layer 6. These results collectively demonstrate that SpaConTDS not only performs consistently across different sequencing platforms but also learns biologically meaningful representations that effectively generalize to downstream analytical tasks.

### SpaConTDS facilitates the study of tumor microenvironment in human breast cancer

**Fig 2:**
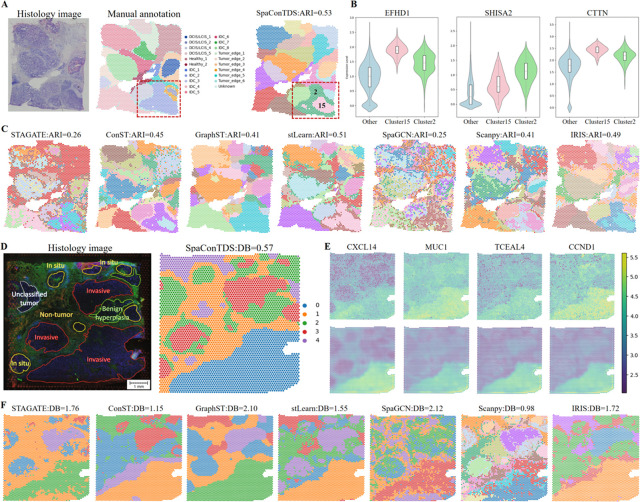
Advanced spatial analysis by SpaConTDS reveals detailed spatial stratification for human breast cancer. (**A**) H&E stained image, manual annotations and clustering result with ARI of SpaConTDS on human breast cancer dataset. (**B**) Violin plots of expression of DEGs (EFHD1, SHISA2, CTTN) in subcluster 2 and 15 versus other clusters. (**C**) Clustering results with ARI of baseline methods on human breast cancer dataset. (**D**) H&E stained image with manually annotated regions and clustering result with DB index of SpaConTDS on IDC dataset. (**E**) Top: raw gene expression patterns of CXCL14, MUC1, TCEAL4andCCND1. Bottom: denoised gene expression patterns of CXCL14, MUC1, TCEAL4andCCND1. (**F**) Clustering results with DB index of baseline methods on IDC dataset.

Breast cancer remains a leading cause of cancer-related morbidity and mortality in women worldwide, with its complex tumor microenvironment (TME) critically influencing tumorigenesis, progression, and therapeutic response. To investigate spatial heterogeneity across breast cancer subtypes (including invasive ductal carcinoma and papillary carcinoma), we applied SpaConTDS to a human breast cancer dataset [30]. Our analysis revealed that SpaConTDS identified spatial domains showed superior concordance with pathological annotations, achieving the highest ARI (ARI = 0.53; Figs 2A, 2C, and S5) among state-of-the-art methods. Of particular note, SpaConTDS uncovered previously unrecognized spatial substructure within region ‘IDC_2’, resolving it into two distinct sub-clusters (sub-clusters 2 and 15; red box, Fig 2A) that may represent functionally distinct tumor niches. As observed in Fig 2B, although both sub-clusters 2 and 15 share common differentially expressed genes (DEGs) involved in tumor invasion and migration (EFHD1 Fig 2B left), these genes exhibit markedly distinct expression patterns between the subclusters (SERHL2, CRISP3 S5C Fig), suggesting fundamental differences in their tumor biology. Furthermore, sub-cluster 2 demonstrates significant upregulation of cell proliferation- and migration-related genes (SHISA2 Fig 2B middle; DUSP23, EIF3H, UBE2S S5C Fig), consistent with an aggressive, fast-growing phenotype. In contrast, sub-cluster 15 shows elevated expression of survival- and resistance-associated gene (CTTN Fig 2B right;IGFBP5, NUPR1, COX7C S5C Fig), indicative of a treatment-resistant subpopulation with enhanced metastatic potential. Taken together, through the multimodal features from SpaConTDS and tumor biological information, We preliminarily suggest dissecting the region’IDC_2’ into ‘Invasive-Proliferative subtype’ and ‘Drug-Resistant and Pre-Metastatic subtype’.

To further investigate the spatial architecture of Invasive Ductal Carcinoma (IDC), we applied SpaConTDS to an IDC-specific dataset [16], results are shown in Figs 2D, 2E, 2F and S5. Davies-Bouldin (DB) index (DB=0.57) demonstrated that SpaConTDS achieved optimal cluster compactness and separation, and visualization of spatial domains depicted consistency with histology images annotated by pathologists. Notably, while competing algorithms erroneously merged ‘’unclassified tumor” regions or major “non-tumor areas" with adjacent tumor regions, SpaConTDS was the only method that accurately preserved these critical anatomical boundaries (Fig 2F), showing remarkable concordance with pathological gold-standard annotations (Fig 2D).

To further characterize the molecular signatures underlying the spatial domains identified by SpaConTDS, we performed comprehensive differential gene expression analysis. The tumor-associated genes such as MUC1, TCEAL4, CCND1, and CXCL14 were highly expressed in cluster 0 (Fig 2E), confirming that the tumor regions identified by SpaConTDS were highly consistent with the true tumor regions. Additionally, the denoised gene expression patterns from SpaConTDS exhibited three key advantages: (1) sharper spatial boundaries between tumor and non-tumor regions, (2) improved spatial continuity within tumor subregions and (3) stronger concordance with pathologist annotations.

These results collectively demonstrate that SpaConTDS effectively extracts biologically meaningful features specific to tumor regions, enabling precise characterization of the tumor microenvironment. It’s capability to resolve complex tumor substructure facilitates a systematic investigation of tumor heterogeneity, the identification of spatially restricted biomarkers, and the discovery of microenvironment-specific therapeutic targets. By providing high-resolution spatial and molecular insights, SpaConTDS emerges as a powerful tool for advancing precision oncology research.

### SpaConTDS can integrate multiple tissue slices without prior spatial alignment

Current spatial transcriptomics studies face inherent technical constraints due to limited tissue section sizes, necessitating division of experimental samples into multiple horizontal or vertical slices for comprehensive regional analysis. A critical preprocessing challenge involves the integration of these discrete tissue slices to reconstruct complete spatial transcriptomic profiles and generate unified gene expression atlases. This requirement for multi-slice joint modeling and analysis remains a fundamental computational challenge in spatial transcriptomics research [19], with important implications for data interpretation and biological discovery.

**Fig 3:**
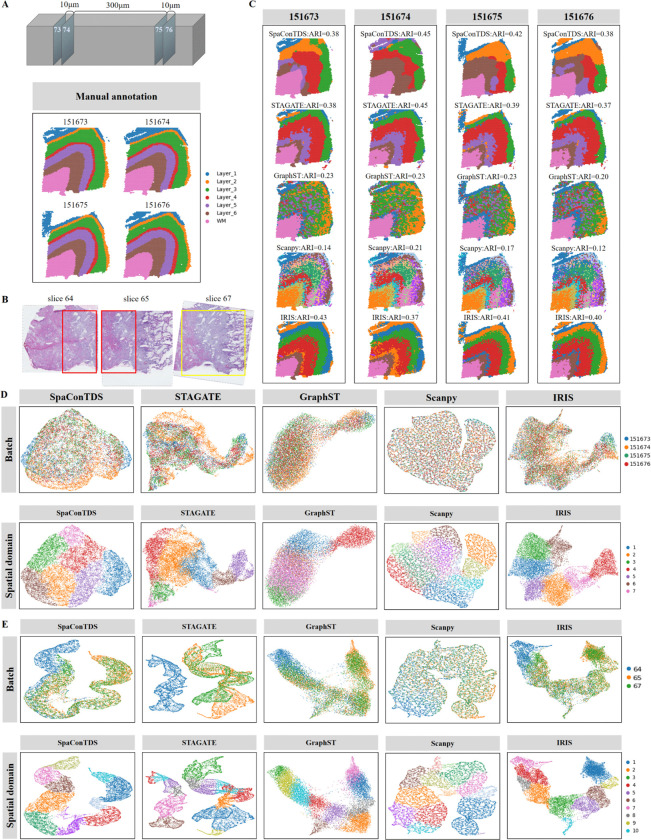
Integration abilities of SpaConTDS. (**A**) Slice sampling diagram and manual annotations of DLPFC slice 151673, 151674, 151675 and 151676. (**B**) Overlapping diagram of three partially overlapping slices from the human placental bed dataset. (**C**) Clustering results with ARI of SpaConTDS, STAGATE, GraphST, Scanpy and IRIS on four DLPFC slices. (**D**) Vertical integration results of DLPFC slices. UMAP plots after batch effect correction (top) and spatial clustering results(bottom) from SpaConTDS, STAGATE, GraphST, Scanpy and IRIS. (**E**) Partial overlap integration results of human placental bed dataset. UMAP plots after batch effect correction (top) and spatial clustering results (bottom) from SpaConTDS, STAGATE, GraphST, Scanpy and IRIS.

To evaluate SpaConTDS’s capability for vertical slice integration, we analyzed 4 consecutive DLPFC slices (151673, 151674, 151675, and 151676) from a single donor, comparing two adjacent pairs (10 μm spacing) separated by 300 μm (Fig 3A). As shown in Fig 3D, SpaConTDS achieved superior integration performance, producing precise alignment across all slices with clear inter-layer boundaries, and significantly improved spatial domain identification accuracy compared to other methods (Fig 3C). Result shows that SpaConTDS effectively integrated these four slices, and generated clear, well-ordered separations between layers (S6A Fig). For the slices that were only 10 μm apart (151675 and 151676), SpaConTDS achieved near-perfect registration, even for the distant slices (300 μm spacing), it maintained a strong alignment (S6A Fig). Only IRIS achieved performance comparable to SpaConTDS. While STAGATE achieved competitive slice-specific ARI scores and spatial domain visualization, it failed to adequately remove batch effects, compromising cross-slice integration. Although GraphST successfully merged those four slices, it showed inadequate separation of layers in UMAP space, and failed to clearly distinguish cortical layers (Fig 3D), indicating limited biological interpretability of its integrated solution.

To further assess the performance of integrating partially overlapping tissue slices, we analyzed a human placental bed dataset comprising three consecutive, biologically distinct regions from a single donor: Myometrium, Decidua, and Placenta [31] (Fig 3B and 3E). Fig 3B reveals that slice 64 and slice 65 share approximately 50% overlap (red box), while slice 65 and slice 67 exhibited over 90% shared tissue area (yellow box). These anatomical relationships were accurately captured by SpaConTDS and GraphST in there batch-effect-corrected embeddings (Fig 3E, “Batch" row), as evidenced by the two facts that partial co-localization (∼ 50%) of blue (slice 64) with yellow/green points (slice 65/67), and limited non-overlap of yellow points in the upper-right UMAP region. Although IRIS achieved comparable overall performance, it was less effective at capturing the slice-specific yellow cluster in the upper-right region. Notably, comparative methods demonstrated contrasting limitations. On one hand, STAGATE under-corrected batch effects, artificially separating slice 64 from slices 65 and 67 into distinct distributions, while, Scanpy over-corrected batch effects, erroneously suggesting complete distributional overlap across all slices. Consequently, it can be inferred that SpaConTDS demonstrated exceptional integration capability, successfully harmonizing the multi-slice dataset while effectively correcting batch effects remarkably. On the other hand, row “Spatial domain" presents UMAP visualization for spatial domains, and it can be revealed that Clusters 7 and 9 are slice-specific regions (unique to slice 64), but they failed to be accurately identified by Scanpy. These results highlight SpaConTDS’s unique ability to simultaneously correct technical artifacts while preserving biological signals, maintain tissue heterogeneity across integration boundaries and retain the molecular identity of unique spatial domains.

### SpaConTDS exhibits robustness and scalability in high-resolution ST data

Heterogeneity in experimental platforms introduces substantial variability in key technical parameters including spatial resolution, gene detection efficiency, and sample processing protocols. These platform-specific characteristics significantly influence the performance of spatial transcriptomics analysis algorithms, necessitating careful methodological consideration when comparing or integrating datasets generated across different technologies [32]. Building upon our previous demonstration of SpaConTDS’s cross-platform generalizability (Result 1), we further evaluated its performance on high-resolution data from the 10X Genomics Xenium platform [13]. This platform provides sub-celluar spatial resolution enabling precise localization of transcriptomic signals and enhanced sensitivity for low-abundance transcripts. While these advancements offer unprecedented biological detail, they simultaneously introduce higher noise and computational challenges in spatial pattern recognition.

**Fig 4:**
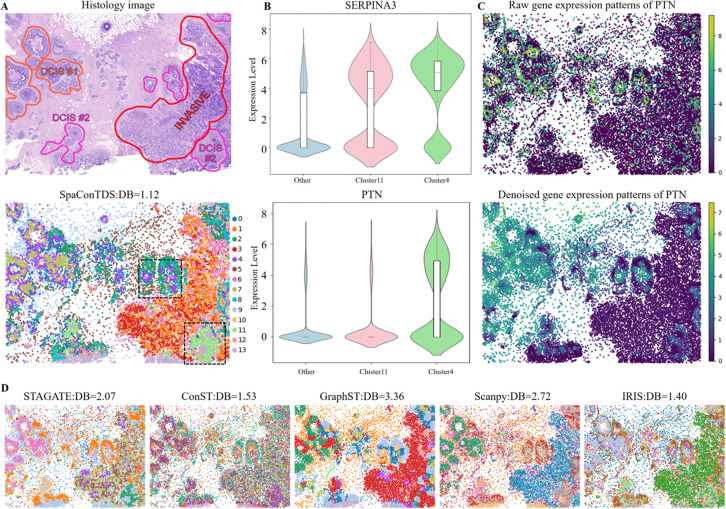
SpaConTDS achieves superior clustering performance on breast cancer 10x Xenium data. (**A**) H&E stained image with manually annotated regions and clustering result with DB index of SpaConTDS on 10x Xenium breast cancer dataset. (**B**) Violin plots of expression of DEGs (SERPINA3, PTN) in subcluster 4 and 11 versus other clusters. (**C**) Raw(top) and denoised(bottom) gene expression patterns of PTN. (**D**) Clustering results with DB index of STAGATE, ConST, GraphST, Scanpy and IRIS.

Fig 4A presents a visualization of (top) pathologist-annotated hstological regions and (bottom) spatial clustering results generated by SpaConTDS. Compared to Fig 4D, it can be concluded from DB index that SpaConTDS achieved superior performance (DB=1.12), and was uniquely capable of accurately identifying the ductal carcinoma in situ(DCIS, labeled as DCIS#1 and #2), especially DCIS#2 region (Fig 4A). While GraphST demonstrated reasonable performance in detecting invasive tumor regions, it misclassified the DCIS#2 region (upper left) as invasive carcinoma. Similarly, Scanpy showed partial success by correctly identifying DCIS#1 but completely failed to delineate DCIS#2 (Fig 4D). These results highlight SpaConTDS’s exceptional precision in distinguishing between in situ and invasive tumor components, a critical capability for clinical diagnostics and tumor margin assessment.

Interestingly, our analysis revealed finer substructure within the DCIS regions, with DCIS#2 further be divided into two distinct subclusters (Cluster 4 and Cluster 11 (black box in Fig 4A)). To validate this subdivision, the DEGs of the DCIS#2 region was visualized in Fig 4B. PTN(a tumor growth and proliferation-related gene) showed significant upregulation in Cluster 4 (*p*<0.01), while no significant differences were observed between Cluster 11 and others. Meanwhile, SERPINA3 (an immune regulator associated with local inflammation) was ubiquitously expressed across the whole DCIS#2 region (*p*<0.01) but exhibited significant expression level differences between clusters. Additionally, we found that the denoised gene expression patterns from SpaConTDS exhibited enhanced spatial continuity within regions, improved alignment with histology images, and sharper contrast between cluster-specific expression profiles (Fig 4C).

These results collectively demonstrate that SpaConTDS is not only robust across multiple spatial transcriptomics platforms and resolution scales but also excels at reconstructing spatially continuous gene expression patterns with high fidelity. By effectively integrating multimodal data including gene expression profiles, spatial coordinates, and histology images, SpaConTDS maintains exceptional precision in spatial pattern recognition even with increasing data complexity. SpaConTDS’s ability to denoise data while preserving biologically meaningful signals enables the identification of distinct spatial domains and molecular gradients, providing clinically actionable insights for tumor subtyping, microenvironment analysis, and therapeutic target discovery. This unique combination of cross-platform compatibility, precise spatial reconstruction, and multimodal integration positions SpaConTDS as a powerful computational framework for advancing spatial transcriptomics research and its translational applications in precision medicine.

### SpaConTDS identifies fine-scale regions in proximal Zebrafish Melanoma Tissue

**Fig 5:**
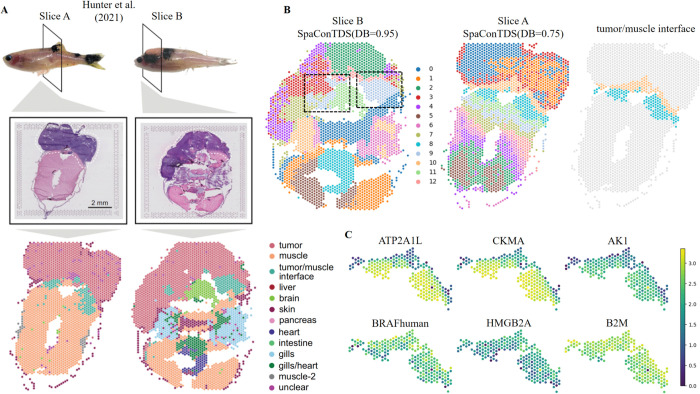
SpaConTDS enhances resolution of interface dynamics in zebrafish melanoma. (**A**) H&E stained image and manual annotations of zebrafish melanoma on slices A and B from [3] with no changes made, and this article is licensed under a Creative Commons Attribution 4.0 international license. (**B**) Interface domains identified by SpaConTDS on slices A and B with DB index. (**C**) The expression of select marker genes(muscle(top): ATP2A1L, CKMA, AK1; tumor(bottom) : BRAFhuman, HMGB2A, and B2M).

The 6.5 mm2 capture area of 10x Visium arrays is particularly well-suited for studying adult zebrafish (∼5 mm diameter), allowing complete transverse sections to be analyzed intact on a single array. This unique advantage enables examination of tumors and their surrounding tissue microenvironments in their native spatial context, avoiding artifacts introduced by tissue dissection or multi-section integration. Preservation of tissue integrity is crucial for studying tumor and its interactions with surrounding tissues, as it effectively eliminates spatial information loss and errors introduced by section integration. Here, we used two zebrafish melanoma slices (A and B) to evaluate the ability of SpaConTDS to resolve fine-scale regional distinctions at tumor margins. The previous study by Miranda V. Hunter et al. suggested that there exists a “transitional" interface region between tumor and normal tissue at the tumor-adjacent microenvironment boundary (Fig 5A, white dotted area). This intermediate region morphologically resembles muscle tissue but exhibits gene expression patterns more similar to the tumor, and it is classified into muscle interface and tumor interface depending on the proximity to the tumor [3].

SpaConTDS outperformed benchmark methods by achieving optimal DB indices (slice A: 0.75; slice B: 0.95) and successfully identifying the transitional interface region that eluded detection by most comparative methods (Figs 5B,S7 top).

The spatial domains detected by the SpaConTDS performed precise alignment with histological annotations, specially, in slice A, Cluster 8 and Cluster 10 accurately delineated the muscle interface and tumor interface subregions respectively, while Cluster 9 in slice B precisely marked the transitional zone. In slice A, Cluster 10 (tumor-proximal interface) showed marked over-expression of established tumor-associated genes including BRAFhuman, HMGB2A, and B2M (Fig 5C bottom), consistent with its anatomical proximity to the tumor mass. This molecular profile reflects paracrine-mediated reprogramming, whereby tumor-derived factors (e.g., proliferative signals, survival factors, and immune modulators) induce an epithelial-mesenchymal transition (EMT)-like state in adjacent tissues. The resulting molecular convergence between Cluster 10 and the tumor core, characterized by shared expression of invasion-related genes suggests active participation of the interface region in facilitating tumor progression through microenvironmental remodeling, enhanced stromal-tumor crosstalk and creation of a pro-invasive niche.

In contrast to the tumor-like molecular profile of Cluster 10, Cluster 8 exhibited significantly elevated expression of muscle-specific genes (AK1, ATP2A1L, CKMA; Fig 5C top), reflecting preserved myogenic function in region.

Furthermore, the spatial transcriptomic pattern reveals a clear gradient of tumor microenvironment influence, where increasing distance from the tumor core correlates with diminished exposure to tumor-derived factors, thus causing maintenance of normal muscle tissue in both morphology and molecular features. The gene expression differences in these areas also reflect the dynamic influence of the tumor microenvironment on adjacent tissues and the varying response patterns of cells to external signals across different regions.

The consistent identification of interface regions in slice B (S7 Fig bottom) further confirms SpaConTDS’s exceptional ability to delineate subtle tumor-stroma boundaries at cellular resolution. By revealing previously overlooked tumor substructures and their spatial relationships with surrounding tissues, SpaConTDS enables more precise histological annotation of zebrafish melanoma samples. Importantly, this high-resolution spatial mapping, when integrated with molecular profiling data, provides a powerful approach to accurately define tumor invasion fronts, quantify microenvironmental remodeling, and assess metastatic potential based on distinct invasive niche characteristics, which may offer new insights into tumor progression mechanisms that could inform therapeutic strategies.

### Effectiveness and importance of image feature extraction

**Fig 6:**
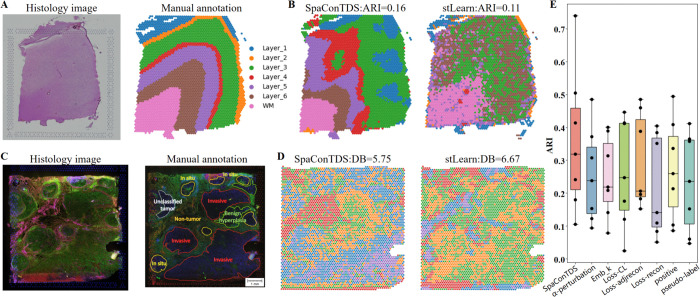
SpaConTDS improves spatial transcriptomic analysis through each module. (**A**) H&E stained image and manual annotations of DLPFC slice 151673. (**B**) Clustering results with ARI based purely on image features by SpaConTDS and stLearn on DLPFC slice 151673. (**C**) H&E stained image and manual annotations of IDC dataset. (**D**) Clustering results with DB based purely on image features by SpaConTDS and stLearn on IDC dataset. (**E**) Boxplot of ARI using SpaConTDS and SpaConTDS without each module on all 7 HER2+ sections.

To specifically evaluate the image feature extraction capability of SpaConTDS, we conducted a comparative analysis using only histology image data from DLPFC slice 151673. Clustering performance assessment demonstrated that SpaConTDS outperformed stLearn (ARI: 0.16 vs 0.11; Fig 6B), indicating that SpaConTDS’s superior ability to extract biologically meaningful features from images. While both methods showed limitations in fully resolving all cortical layers, SpaConTDS exhibited two key advantages: (1) precise identification of white matter (WM) and layer 6 that closely matched manual annotations, and (2) preservation of critical textural patterns that reflect true spatial organization. In contrast, stLearn failed to capture most laminar features except for a rudimentary WM detection. Although SpaConTDS’s layer 1-4 discrimination remained suboptimal, its partial success in layer-specific feature extraction suggests that histological patterns contain detectable but complex signatures of cortical lamination that conventional methods fail to adequately utilize.

Given the inherent limitations of DLPFC (limited image information and substantial noise), we further evaluated SpaConTDS’s feature extraction capability using H&E image from IDC dataset (Fig 6D). While both methods showed suboptimal performance on the DB index, (SpaConTDS, DB=5.75; stLearn, DB=6.67), reflecting the challenge of tumor region segmentation from histology image alone, SpaConTDS demonstrated more accurate identification of invasive tumor margins and unclassified tumor regions. These results indicate that SpaConTDS effectively extracts discriminative histological features, and more importantly, demonstrate that the integration of these image-derived features with transcriptomic data (as shown in previous analyses) is essential for achieving optimal spatial domain recognition performance in complex tissue architectures.

Additionally, our analysis revealed a significant correspondence between SpaConTDS-derived cluster boundaries and histologically visible tissue structures in H&E images (Fig 6D), demonstrating the method’s effectiveness in capturing biologically relevant architectural patterns. This observation highlights two critical considerations for multimodal integration: the need to optimally weight image-derived features in the global representation to mitigate noise interference, and the importance of our proposed adaptive hyperparameter *k*_*emb*_ in dynamically balancing feature contributions. These findings collectively underscore that while histological features provide valuable structural information, their judicious integration with transcriptomic data, mediated by appropriate weighting mechanisms, is essential for achieving robust spatial domain characterization.

To systematically evaluate the contribution of each component in SpaConTDS, we performed comprehensive ablation studies using the HER2ST dataset. Fig 6B indicates that each modality contributes valuable information. Ignoring any modality leads to a significant drop in the performance of SpaConTDS, thereby confirming the importance of multimodality. Moreover, in Fig 6E, removing the hyperparameter *Emb*_*k*_ also causes a performance decline, suggesting that, in addition to multimodal fusion, the contribution of each modality must be taken into account, rather than simply performing a straightforward summation.

The loss function used in this paper consists of three components: the contrastive loss function (*Loss*_*CL*_), the graph connection prediction loss function (*Loss*_*adjrecon*_), and the gene expression reconstruction loss function (*Loss*_*recon*_). In ablation study, we found that removing any of these three loss functions leads to a decline in performance (Fig 6E), demonstrating that all three parts are necessary.

In positive sample construction section, we innovatively enhanced the positive samples for the gene modality by utilizing neighbor information. Therefore, ablation experiments, we only retained conventional augmentation methods, such as adding noise. Fig 6E shows a decline in performance, indicating the effectiveness of the gene modality enhancement approach.

For the negative sample construction in ablation experiments, we performed the following operations. in ‘pseudo-label’ case, we performed a tuple disturbing strategy by shuffling the sample order instead of pseudo-labels; in ‘*α*-perturbation’ case, we fixed alpha to 1/3, avoiding adaptive adjustment, thereby perturbing all modalities with equal probability. The performance of SpaConTDS declined in both scenarios (Fig 6E). These results underscore the critical importance of all modules and modalities utilized in SpaConTDS for overall effectiveness, as well as the coordinated interaction between different modules and modality information.

## Discussion

In-depth exploration of the multimodal information within Spatial Transcriptomics (ST) data is essential for understanding the heterogeneity of tissue structure, investigating biological functions and tracking disease progression. However, ST faces several technical limitations that require analytical algorithms to mitigate. Firstly, the capture area of ST is size-limited, requiring the integration of multiple tissue sections to capture a larger tissue sample. Secondly, data from different platforms possess distinct characteristics, such as varying resolutions, necessitating algorithms with a certain degree of generalizability to process data with diverse formats and resolutions.

In this paper, we presented a method SpaConTDS, based on multimodal contrastive learning and reinforcement learning, which integrates gene expression and histopathological image information for spatial domain identification, alignment-free slice integration and various downstream tasks. Compared to the existing benchmarks, SpaConTDS showed improved clustering accuracy on ST data of different resolutions and platforms (10x Visium, 10x Xenium, and ST) and identified finer tissue structures. Furthermore, SpaConTDS can recognize biologically coherent spatial domains in aligned samples and effectively remove batch effects when integrating multiple slices.

The key to SpaConTDS responsible for its superior performance lies in the construction of positive/negative samples through augmentation and a pseudo-label tuple perturbation strategy, which aids in learning fused representations containing global semantics and interactions between modalities. Although existing methods, such as ConGI, also utilize multimodal contrastive learning, they only perform linear weighting of the representations of different modalities and do not account for interactions between modalities, which hampers their performance. SpaConTDS differs in the construction of positive/negative samples, modality encoders, objective functions and contrastive loss formulas, as well as incorporates reinforcement learning during training. These differences allow SpaConTDS to outperform ConGI in clustering and make it applicable to a wide range of data types and analytical tasks. We also validated the effectiveness of these differences and individual modules within SpaConTDS through ablation experiments.

In future work, we aim to extend SpaConTDS in several directions. First, we plan to expand it to more ST platforms such as Stereo-seq [33], Slide-seqV2 [12], MERFISH [7], and Nanostring CosMx SMI [34] to assess SpaConTDS’s performance on different data formats and datasets lacking image information. Additionally, some existing methods, such as HisToGene [35] and Hist2ST [36], can predict gene expression using only H&E stained images. Therefore, enhancing SpaConTDS’s feature extraction ability of H&E stained image to perform accurate spatial domain identification solely based on image information is both feasible and promising. Furthermore, the future development in ST technologies may bring about subcellular resolution data with full gene expression profiling, meaning an increased number of spots in ST datasets, which would result in higher GPU memory consumption. The GraphSAGE and Hist2ST convmixer used by SpaConTDS require storage of intermediate computational results (e.g., node features, adjacency matrices, convolutional feature maps) on the GPU, and the neighbor sampling and aggregation operations of GraphSAGE may involve irregular memory access patterns. These all result in high time complexity and GPU memory usage. Therefore, in future work, we consider to apply graph sparsification by retaining only the important edges [37] and utilize a lightweight model (e.g., shallow GNN [38] or small CNN [39]) to perform knowledge distillation from the complex model [40], thereby optimizing the computational efficiency of SpaConTDS.

## Materials and methods

In this section, we present the framework for SpaConTDS. More implementation details can be found in S1 Text Sect 3.

### Overview of SpaConTDS

**Fig 7:**
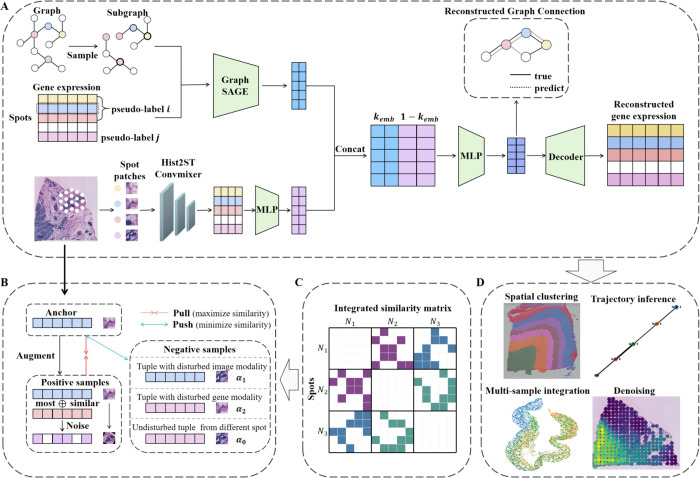
Schematic overview of SpaConTDS. (**A**) Framework of SpaConTDS. (**B**) Overview of the positive and negative samples construction strategy. (**C**) For multiple tissue slices, SpaConTDS constructs a global similarity matrix across all slices to generate positive samples with cross-slice information. (**D**) Downstream tasks.

SpaConTDS is a self-supervised multimodal contrastive learning framework comprising a multimodal encoder, a contrastive learning (CL) module and a reinforcement learning (RL) module. An overview of the pipeline is presented in Fig 7. The multimodal encoder and CL module are designed to jointly learn spot representations from input gene expression profiles, spatial coordinates and histology images. The RL module is employed to adaptively optimize hyper-parameters, thereby enhancing the robustness and accuracy of the model.

The multimodal encoder of SpaConTDS takes gene expression, spatial coordinates, and histology images as input to generate fused spot representations that capture the interactions among modalities, which can be subsequently utilized to infer spatial domain structures, as seen in Fig 7A. Specifically, a spatial neighborhood graph is first constructed, in which spatially adjacent spots are connected. GraphSAGE [41] is then employed as the encoder for the gene modality, embeding both gene expression and spatial similarity into a latent feature space. Concurrently, histology images are segmented into patches aligned with the spatial location and size of each spot, and then Hist2ST convmixer [36] is applied to extract image features corresponding to each spot. Finally, a multilayer perceptron(MLP) [42] is used to fuse features from different modalities, weighted according to their relative contributions. The resulting fused representation effectively captures inter-modality interactions.

The CL module operates within the fused representation space, aiming to obtain embeddings that encapsulate inter-modal interactions and global semantic features. This is achieved by increasing the similarity between anchors and positive samples while simultaneously decreasing the similarity between anchors and negative samples (Fig 7B). To mitigate the potential underutilization of weak modalities (such as histology images), SpaConTDS introduces a tuple disturbing strategy based on TupleInfoNCE [43], which constructs negative samples using pseudo-labels. And it can be proved that the tuple disturbing strategy and TupleInfoNCE can effectively prevent the neglect of weak modalities and learn the interactions between modalities, see S1 Text Sect 6 for details. The learning process jointly optimizes three loss components: graph-connection reconstruction loss, feature reconstruction loss and contrastive loss. The joint objective enhances the spatial smoothness, sematic richness and discriminative power of the learned representations.

In addition to processing individual tissue slices, SpaConTDS is also capable of handling horizontal, vertical and partially overlapping slice integration (Fig 7C). Prior to training, histology images from multiple slices are concatenated, and a global similarity graph is constructed to capture inter-slice relationships. This graph, along with the concatenated images, is then used as input for feature extraction, enabling SpaConTDS to integrate gene expression, histological and spatial information across multiple slices. In contrast to most existing approaches that rely on prior spatial alignment, SpaConTDS can automatically smooth features between adjacent spots, both within the same slice and across different slices, while simultaneously correcting batch effects during model training. A detailed illustration of each part would be presented as follows.

### Data preprocessing

Preprocessing pipeline for gene expression matrices consisted of three steps: selection of the top 3000 highly variable genes (HVGs), library normalization (set as 10^4^) and log transformation of the normalized gene expression profiles. SCANPY package [44] was used for processing the pipeline and the processed gene expression **X** served as the primary input for SpaConTDS.

As for image modality, histology image patches were extracted based on spatial spots’ coordinates, with patch dimensions standardized to match the spot diameter of each sequencing platform, that is 112 × 112 pixels for ST and 10x Visium platforms and 30 × 30 pixels for 10x Xenium platform.

In the subsequent data augmentation, we need to use the gene expression information of the nearest and the most similar neighbor for each spot to augment the gene modality. Therefore, we constructed an undirected neighborhood graph *G* based on spatial information. Let $A\in {\mathbb{R}}^{N_{\text{spot}}\times N_{\text{spot}}}$ be the symmetric similarity-adjacency matrix of graph *G*. For spot *s*_*i*_, we selected 6 nearest neighbors based on the spatial Euclidean distance (excluding the spot itself). Among these spots, if spot *s*_*j*_ has the most similar gene expression to spot *s*_*i*_, $a_{ij}=a_{ji}=1$, otherwise 0.

### Framework of SpaConTDS

**Multi-modal encoder-decoder.** Multi-modal module is used to learn multimodal fusion representations and reconstruct gene expression containing histological features by decoding the fused features.

**Histology image encoder.** Utilizing pre-trained convolutional neural networks models to extract image features can effectively enhance model performance and stability in data-limited scenarios. However, standard models are typically pre-trained on natural image datasets like ImageNet [45]. The domain shift between natural RGB images and hematoxylin-eosin (H&E) stained tissue sections, characterized by their distinctive purple-red chromatic distributions, substantially reduces feature extraction efficacy. This morphological and chromatic discrepancy suggests that standard pre-trained models may fail to capture biologically relevant patterns in histology images.

In this paper, the histology image encoder was designed based on Hist2ST convmixer [36], which is part of the Hist2ST model, a specialized architecture pretrained for histology images and can independently extract features from individual histology image patch. To enhance model stability and prevent overfitting, the Hist2ST Convmixer network’s parameters were maintained frozen during training, thereby preserving its learned histological representations while preventing gradient updates through backpropagation. Afterwards, a learnable MLP is incorporated after the pretrained Hist2ST convmixer for adaptive feature refinement. Given memory constraints imposed by typical histology datasets, we employed batch processing with batch size *B*. Each forward pass processes *B* image patches through the pretrained Hist2ST Convmixer and the trainable MLP, finally producing image features ${𝐡}_{img}\in {\mathbb{R}}^{B\times d}$, where *d* represents the dimension of the features.

**Spatial transcriptomics encoder.** The spatial transcriptomics data were encoded using a graph neural network to explicitly model both local neighborhood relationship between spots and global pattern of gene expression profiles. An undirected neighborhood graph *G=(V,E)* was constructed based on the spatial information, where *V* represents the set of spots. For each pair of spots $\left(s_{i},s_{j}\right)$, its spatial distance was computed as

$$d(s_{i},s_{j})=\sqrt{(x_{i}-x_{j}{)}^{2}+(y_{i}-y_{j}{)}^{2}}$$ (1)

where $\left(x_{i},y_{i}\right)$ and $\left(x_{j},y_{j}\right)$ denote the spatial coordinates of spot *s*_*i*_ and *s*_*j*_, respectively. Then, for each *s*_*i*_, an undirected edge was established to its k-nearest neighbors (kNN) based on the spatial distances, *k* was set to 6. To maintain consistency with the image modality’s batch training paradigm while ensuring computational efficiency, we implemented a two-layer GraphSAGE encoder for gene expression data. The encoder operates through two steps, hierarchical neighborhood sampling and feature aggregation. Firstly, for each central spot *s*_*i*_, its first-order neighbors and second-order neighbors were uniformly sampled, and let $N^{\left(1\right)}(s_{i})$ ($N^{\left(2\right)}(s_{i})$) denote the first-order (second-order, respectively) sampled neighbors’ set, and $S_{1,2}=N^{\left(1\right)}(s_{i})\;\cup \;N^{\left(2\right)}(s_{i})$ be the corresponding subgraph. Then, representation for spot *s*_*i*_ can be updated through sequential aggregation from outer-most to central node, according to the following formula:

$${𝐡}_{ST_{i}}^{t+1}=\sigma (W_{1}\cdot f_{mean}({𝐡}_{ST_{i}}^{t}\cup \{{𝐡}_{ST_{j}}^{t+1}|j\in N^{\left(1\right)}(s_{i})\}))$$ (2)

$${𝐡}_{ST_{j}}^{t+1}=\sigma (W_{0}\cdot f_{mean}({𝐡}_{ST_{j}}^{t}\cup \{{𝐡}_{ST_{k}}^{t}|k\in N^{\left(2\right)}(s_{i})\cap N^{\left(1\right)}(s_{j})\}))$$ (3)

where, ${𝐡}_{ST_{i}}^{t}$ is the representation for *s*_*i*_ at *t*–th iteration, *W*_0_ and *W*_1_ are the learnable network parameters, σ represents the activation function, $f_{\text{mean}}$ is the mean calculation operator.

**Multimodal representations fusion.** To model cross-modal interactions while preserving modality-specific information, we integrated the spatial gene expression features ${𝐡}_{ST}\in {\mathbb{R}}^{B\times d}$ and histology image features ${𝐡}_{img}\in {\mathbb{R}}^{B\times d}$ through an attention-based fusion mechanism:

$${𝐡}_{c}=f_{concat}(k_{emb}\cdot {𝐡}_{ST},(1-k_{emb})\cdot {𝐡}_{img})$$ (4)

$${𝐡}_{f}=W_{f}\cdot {𝐡}_{c}$$ (5)

where, ${𝐡}_{c}\in {\mathbb{R}}^{2B\times d}$ is a weighted concatenated transition representation for each batch, *f*_*concat*_ is the concatenation operator and $k_{emb}\in (0,1)$ is a dynamic weighting coefficient optimized via reinforcement learning, to adaptively balancing the modality contributions based on their relative information content. ${𝐡}_{f}\in {\mathbb{R}}^{B\times d}$ is the fused representation for each batch, *W*_*f*_ is a trainable projection matrix.

**Spatial smoothness of fused representations.** To preserve the spatial coherence of fused representations, we introduced a graph topological regularization task that reconstructed the original spatial graph *G* from the fused spot representations. Let ${\hat{A}}_{ij}$ denote the adjacency matrix of the reconstructed graph $\hat{G}$, then the reconstruction probability between a spot pair $\left(s_{i},s_{j}\right)$ was modeled as

$$p({\hat{A}}_{ij}=1|{𝐡}_{f_{i}},{𝐡}_{f_{j}})=sigmoid({𝐡}_{f_{i}}\cdot ({𝐡}_{f_{j}}{)}^{T})$$ (6)

where ${𝐡}_{f_{i}}$ and ${𝐡}_{f_{j}}$ are the fused representations of spot *s*_*i*_ and *s*_*j*_, respectively.

Then for each batch, a subgraph *S* was sampled from *G* using GraphSAGE, with adjacency matrix *A*^*S*^. Given the low edge density of the k-NN constructed graph, we restricted connection prediction to positively connected spot pairs $\left\{(s_{i},s_{j})|A_{ij}^{S}=1\right\}$ to avoid imbalance. Then the adjacency reconstruction loss $L_{recon-a}$ could be computed according to the following formula:

$$L_{recon-a}=\sum_{S}\frac{\sum_{i,j}A_{ij}^{S}\cdot [1-p({\hat{A}}_{ij}=1){]}^{2}}{\#(S)}$$ (7)

Here, #(S) denotes the number of edges in subgraph *S*_*b*_.

**Decoder.** The fused representations ${𝐡}_{f}$ integrate transcriptional profiles with histologically derived structural and morphological patterns, capturing critical cell-microenvironment interactions, and reflects the intrinsic mechanism of gene regulation. To leverage these enriched features for gene expression modeling, we employed a two-layer GCN as a decoder. The GCN inverts ${𝐡}_{f}$ back to the original spatial gene expression profiles $\hat{𝐗}$. The reconstruction loss $L_{recon-b}$ can be calculated according to the following formula:

$$L_{recon-b}=\sum_{i=1}^{b}(\sum_{j=1}^{B}||{\hat{𝐗}}_{ij}-{𝐗}_{ij}|{|}_{2}^{2})$$ (8)

Here, *b* denotes the number of batch.

**Contrastive learning module.** Contrastive learning is performed in the feature space derived from the fused modalities. We will introduce how this module effectively prevents the neglect of weak modalities while obtaining fused representations with modality interactions and global semantics, by focusing on data augmentation, the Pseudo-label tuple perturbation strategy, and contrastive loss.

**ST modality augmentation.** Spatial domains in real tissue samples often exhibit strong spatial continuity, wherein spots with proximate coordinates tend to belong to the same domain and display similar gene expression profiles. To leverage this property, beyond conventional noise injection, we proposed an augmentation strategy for ST modality that incorporates gene expression information from each spot’s nearest and most similar neighboring spot. This approach not only smooths the distribution of gene expression features, but also aligns it with the inherent spatial gradient present in ST data. Importantly, it preserves spatial coherence and mitigate the risk of introducing excessive noise or biologically implausible outlier samples.

Formally, for each feature matrix ${𝐡}_{ST}\in {\mathbb{R}}^{B\times d}$ of subgraph *S*_*b*_, where *B* denotes the batch size and *d* represents the dimension of the features, we first constructed an adjacency matrix $A^{b}\in {\mathbb{R}}^{B\times B}$, derived from the symmetric similarity-adjacency matrix *A*. Subsequently, for each spot *s*_*i*_ in the batch *b*, the corresponding augmented feature representation is updated according to the following formula:

$${𝐡}_{ST_{i}}^{+}=\frac{{𝐡}_{ST_{i}}+\frac{\sum_{k=1}^{m_{i}}{𝐡}_{ST_{k}}}{m_{i}}}{2}$$ (9)

Here, ${𝐡}_{ST_{i}}$ is the spatial gene expression features of spot *s*_*i*_, *m*_*i*_ denotes the degree of spot *s*_*i*_ in *S*_*b*_. Furthermore, to account for the various uncontrollable factors such as environmental variations and technical inherent in the spatial transcriptomics sequencing process, which may introduce stochastic fluctuations into the gene expression profiles, we applied noise perturbation to enhance the node feature matrix ${𝐡}_{ST}$. Specifically, among the *d* dimensions of the features, we randomly selected a subset compromising d×mask dimensions and perturb their values by adding Gaussian noise sampled from a normal distribution with zero and unit variance. Finally, perform imputation on the expression matrix by replacing missing values with the row average.

**Image modality augmentation.** For the histology image modality, due to the relatively limited amount of information contained in individual histological patches, directly perturbing the pixel values (e.g., through color jittering or noise addition) may distort the semantic content of the image. To preserve biologically relevance while still introducing variation, we instead applied a series of conservative yet effective augmentations. Specially, each image was subjected to random horizontal and vertical flips with a probability of 30%, followed by Gaussian blurring with a 30% probability to simulate defocus effect. Finally, random rotations were applied to generate the augmented patches, and the augmented patches were input into the histology image encoder to obtain the augmented feature representation ${𝐡}_{img}^{+}$ of the image modality. The augmented features of two modalities were fused through multimodal representations fusion to obtain the positive samples ${𝐡}^{+}$, ${𝐡}_{i}^{+}$ is the positive sample obtained by augmenting the anchor ${𝐡}_{i}$.

**Pseudo-label tuple perturbation strategy.** The core idea of this strategy is to construct k-disturbed negative samples based on pseudo-labels, with the specific procedure outlined as follows. First, clustering was performed on the image features to assign a pseudo-label to each spot. For spot *s*_*i*_, treated as an anchor, is represented as a K-tuple


$${𝐡}_{i}=({𝐯}_{i1},{𝐯}_{i2}),{𝐯}_{i1}=W_{ST}\cdot (k_{emb}\cdot {𝐡}_{ST}),{𝐯}_{i2}=W_{img}\cdot ((1-k_{emb})\cdot {𝐡}_{img})$$

Let *l*_*i*_ be the pseudo-label of the anchor spot, $D=\{{𝐡}_{1},{𝐡}_{2},...,{𝐡}_{B}\}$ represents the sample set for each batch.

From the set *D* excluding ${𝐡}_{i}$, we randomly selected a sample ${𝐡}_{j}=({𝐯}_{j1},{𝐯}_{j2})$, wherej≠i and pseudo-label $l_{j}\neq l_{i}$. To construct a k-disturbed negative sample, we replaced the *k*_*th*_ modality of ${𝐡}_{i}$ with that of ${𝐡}_{j}$, producing


$${𝐡}_{i,j}^{2-}=({𝐯}_{i1},{𝐯}_{j2}),{𝐡}_{i,j}^{1-}=({𝐯}_{j1},{𝐯}_{i2}),j\neq i$$

where the k-th element originates from the mismatched sample ${𝐡}_{j}$, and all other modalities remain from ${𝐡}_{i}$. This results in a semantically corrupted sample that maintains most modality-specific information from the anchor while only introducing inconsistency in one modality, thereby enhancing the model’s ability to learn interactions between modalities.

Intuitively, differing pseudo-labels reflect substantial differences in the organizational structures of the corresponding spots, indicating a high likelihood that they originate from different spatial domains. This effectively mitigates the risk of constructing negative samples from spatially distant spots that nonetheless belong to the same domain, a scenario that could otherwise impair the model’s ability to learn meaningful representations. Furthermore, when the *k*_*th*_ modality corresponds to a weak modality, the anchor sample and its *k*-disturbed counterpart differs only in that specific modality, with all the other modalities remaining identical. In this case, thesole distinguishing factor between the positive sample ${𝐡}_{i}^{+}$ and the k-disturbed negative sample ${𝐡}_{i,j}^{k-}$ lies in the k-th modality. This setup compels the model to capture and utilize the discriminative information present in the weak modality *k*, thereby mitigating the risk of being ignored during training. To control the contribution of each modality-specific disturbance, we introduced learnable hyper-parameter $\alpha_{k}$, which denotes the proportion of *k*-disturbed negative samples and is dynamically updated via reinforcement learning. The resulting negative samples are drawn from a mixture distribution defined as follows,

$${𝐡}_{i,j}^{-}=\alpha_{0}\cdot {𝐡}_{j}+\sum_{k=1}^{2}\alpha_{k}\cdot {𝐡}_{i,j}^{k-}$$ (10)

Here $\alpha_{0}$ denotes the probability of of constructing a fully disturbed negative sample, in which all *K* modalities are replaced by the corresponding modalities from the *j*_*th*_ sample. $\alpha_{k}$ represents the probability that only the *k*_*th*_ modality is replaced by its counterpart from the *j*_*th*_ sample, while the remaining *K*–1 modalities are retained from the *i*_*th*_ sample. Intuitively, assigning a larger value to $\alpha_{k}$ increase the proportion of *k*-disturbed negative samples in training, thereby placing greater emphasis on the model’s ability to extract informative features from the *k*-th modality.

**Contrastive loss.** We used the widely adopted InfoNCE loss function [46] as the contrastive loss:

$$L_{con}=\sum_{j=1}^{b}(\sum_{i=1}^{B}(-E_{\left({𝐡}_{i},{𝐡}_{i}^{+},{𝐡}_{i,j;j\neq i}^{-}\right)}\left[\log\frac{\exp({𝐡}_{i}\cdot {𝐡}_{i}^{+})/\tau }{(\exp({𝐡}_{i}\cdot {𝐡}_{i}^{+})+\exp({𝐡}_{i}\cdot {𝐡}_{i,j}^{-}))/\tau }\right]))$$ (11)

**Overall loss function.** SpaConTDS is optimized by jointly minimizing the graph connectivity reconstruction loss, the gene expression reconstruction loss and the contrastive loss. The overall training losswas formulated as follows:

$$L_{recon}=L_{recon-b}+\gamma_{1}\cdot L_{recon-a}$$ (12)

$$L=L_{con}+\gamma_{2}\cdot L_{recon}$$ (13)

Here, $\gamma_{1}$ and $\gamma_{2}$ are weight factors that balance the impacts of each loss component. Empirically, we set them both as 1.

### Hyper-parameters settings

Reinforcement learning was utilized to adaptively update α. We began by indepedently performing clustering on each unimodal representation. Let *l*^*img*^ and *l*^*ST*^ denote the pseudo-labels obtained from clustering based on histology image features and gene expression features, respectively. To guide the optimization of the modality weighting parameter α, we exploited the consistency between clustering results across modalities. Specifically, α was optimized by maximizing the following reward function:

$$ℛ(\alpha_{i})=\frac{\text{ARI}(l_{\alpha_{i}}^{img},l_{\alpha_{i}}^{ST})-\text{ARI}(l_{\mu_{t}}^{img},l_{\mu_{t}}^{ST})}{\text{ARI}(l_{\mu_{t}}^{img},l_{\mu_{t}}^{ST})}$$ (14)

Specificlly, $l_{\alpha_{i}}^{img}$ (or $l_{\mu_{t}}^{img}$) denotes the pseudo-labels of spatial domains obtained from histological modality under a setting of hyper-parameter α ($\mu_{t}$, respectively). $l_{\alpha_{i}}^{ST}$ (or $l_{\mu_{t}}^{ST}$) denotes the pseudo-labels of spatial domains obtained from spatial transcriptomics modality under a setting of hyper-parameter α ($\mu_{t}$, respectively). Moreover, hyper-parameter α is randomly generated from $\alpha_{i}\sim 𝒩(\mu_{t},\sigma I)$. ARI function is used to measure the consistency of pseudo-labels between different modalities. The reward function used to evaluate the embeding-specific hyper-parameter *k*_*emb*_ follows the same formulation as described above.

We integrated the optimization of hyper-parameters and the model **g** in a unified framework, as summarized in **Algorithm 1**. Specially, we adopt an alternating optimization strategy, where the hyper-parameters and the model **g** are updated in turn within a single training pass. Other hyperparameter settings are provided in the S1 Text Sect 3.

**Algorithm 1 Hyper-parameter optimization.**

**Input:** Initialized model ${𝐠}_{0}$, initialized distribution $\left(\mu_{0}^{\alpha },\sigma^{\alpha }\right)$ and $\left(\mu_{0}^{k},\sigma^{k}\right)$, total training epochs *T*, distribution learning rate *η*

**Output:** Final model ${𝐠}_{T}^{\alpha^{*}k^{*}}$

  **for** *t* = 1 to *T* **do**

   **if** *t* is even **then**

    Sample *B* **sampling ratio** hyper-parameters $\{\alpha_{i}{\}}_{i=1}^{B}$ via distribution $𝒩(\mu_{t}^{\alpha },\sigma^{\alpha }I)$;

    Train ${𝐠}_{t}$ for one epoch separately with each $\alpha_{i}$ and get $\{{𝐠}_{t+1}^{i}{\}}_{i=1}^{B}$;

    Calculate rewards $\{ℛ(\alpha_{i}){\}}_{i=1}^{B}$, using Equation 14;

    Decide the best model $j=\arg\max_{i}ℛ(\alpha_{i})$;

    Update $\mu_{t+1}^{\alpha }=\mu_{t}^{\alpha }+\eta \frac{1}{B}\sum_{i=1}^{B}ℛ(\alpha_{i})\nabla_{\alpha }\log(p(\alpha_{i};\mu^{\alpha },\sigma^{\alpha }))$;

    Update ${𝐠}_{t+1}={𝐠}_{t+1}^{j}$;

   **else**

    Sample *B* **fused weight** hyper-parameters $\{k_{i}{\}}_{i=1}^{B}$ via distribution $𝒩(\mu_{t}^{k},\sigma^{k}I)$;

    Train ${𝐠}_{t}$ for one epoch separately with each *k*_*i*_ and get $\{g_{t+1}^{i}{\}}_{i=1}^{B}$;

    Calculate rewards $\{ℛ(k_{i}){\}}_{i=1}^{B}$, using Equation 14;

    Decide the best model $j=\arg\max_{i}ℛ(k_{i})$;

    Update $\mu_{t+1}^{k}=\mu_{t}^{k}+\eta \frac{1}{B}\sum_{i=1}^{B}ℛ(k_{i})\nabla_{k}\log(p(k_{i};\mu^{k},\sigma^{k}))$;

    Update ${𝐠}_{t+1}={𝐠}_{t+1}^{j}$;

   **end if**

  **end for**

  **return** ${𝐠}_{T}$

### Integration of slices

**Image and spatial coordinate processing.** Histology images from multiple slices are horizontally concatenated, and the spatial coordinates of each slice are translated to align with the pixel positions in the concatenated image. This pre-processing step ensures that, during multi-slice integration, each spot retains accurate spatial reference for patch extraction, without coordinate collisions across slices. As a result, the concatenated image and the integrated dataset can be directly used as inputs to SpaConTDS, without any additional spatial alignment procedures.

**Similarity matrix.** For the integrated dataset comprising *n* slices with respective number of spots $N_{1},N_{2},...,N_{n}$, we constructed a similarity matrix $A^{*}\in {\mathbb{R}}^{T\times T}$, where the total number of spots $T=\sum_{i=1}^{n}N_{i}$. Specifically, for any spot *s*_*i*_ in slice *S*_*i*_, we identified the top *k* most similar spots from every other slice *S*_*j*_,*j* = *i* + 1,...,*n*. Bidirectional connections are then established between *s*_*i*_ and the selected spots to construct the inter-slice similarity matrix A^*^. All subsequent operations follow the same protocol as in the single slice setting.

This cross-slice construction effectively mitigates batch effects between adjacent tissue sections and ensures that both positive and negative sample selection for CL module spans across all *n* slices. It facilitates the alignment of spatially and biologically adjacent spots across slices, while simultaneously encouraging separation between non-adjacent or pseudo-label-inconsistent spots. In our implementation, the hyperparameter *k* is set to 6.

### Spatial domain assignment via clustering and refinement

For datasets with manual annotations, the number of clusters is set to match the number of ground-truth classes. We employed Leiden algorithm for clustering, using a binary search to identify the resolution parameter that yields the number of clusters. The search is terminated either when the target number of clusters is achieved or after a maximum of 50 iterations. In the absence of manual annotations, we applied Leiden with a range of resolution values from 0.1 to 3.0, with an increments of 0.05. The resolution yielding the highest Structural Clustering (SC) score is selected.

SpaConTDS additionally incorporates an optional refinement step designed to further reduce noise and promote smoother cluster boundaries. Specifically, for a given spot *s*_*i*_, all neighboring spots within a pre-defined spatial radius *r* are identified, and spot *s*_*i*_ was reassigned to the most frequent cluster label among its neighbors.

This step is not recommended for datasets exhibiting fine-grained structures, such as the anterior and posterior regions of the mouse brain, where local smoothing may obscure meaningful boundaries . In this study, we applied this refinement procedure only to the HER2+, DLPFC and human breast cancer datasets, where broader spatial domains benefit from enhanced boundary regularization.

### Downstream analysis

The learned representations are applicable to various downstream tasks.

**Differentially expressed genes(DEG) analysis.** For spatial clusters identified by SpaConTDS, we used the Scanpy package to compute the expression differences of each gene between the target cluster and other clusters. Genes that are significantly upregulated in the target cluster, based on their computed p-values, were identified as differentially expressed genes (DEGs) for that specific region.

**Trajectory inference via PAGA.** Partition-based Graph Abstraction (PAGA) is applied to identify cellular trajectories by constructing a graph where nodes represent identified clusters and edges reflect potential transitions between these clusters. Here, we utilized PAGA which is implemented in the Scanpy package ‘sc.tl.paga’.

**UMAP visualization.** UMAP is a powerful tool, which reduces the high-dimensional expression data to 2D for visualization while preserving both local and global structure. This allows us to visually explore the clustering results and the spatial organization of cells, as well as observe how these clusters relate to each other. Here, we applied UMAP using ‘sc.tl.umap’ in Scanpy package.

## Supporting information

S1 Text Supplementary note. (PDF)

S1 Table Description of all ST datasets used in SpaConTDS. (PDF)

S1 Fig Manual annotations and comparison of spatial domains identified by SpaConTDS, STAGATE, ConST, GraphST, stLearn and SpaGCN on the 7 sections of HER2-positive breast tumor (HER2+) dataset with ARI and NMI. (PDF)

S2 Fig Manual annotations and comparison of spatial domains identified by SpaConTDS, scanpy, Louvain, ConGI, IRIS, Miso, and MorphLink on the 7 sections of HER2-positive breast tumor (HER2+) dataset with ARI and NMI. (PDF)

S3 Fig Manual annotations and comparison of spatial domains identified by SpaConTDS, STAGATE, ConST, GraphST, stLearn and SpaGCN on the 12 slices of DLPFC dataset with ARI and NMI. (PDF)

S4 Fig Manual annotations and comparison of spatial domains identified by SpaConTDS, scanpy, Louvain, ConGI, IRIS, Miso and MorphLink on the 12 slices of DLPFC dataset with ARI and NMI. (PDF)

S5 Fig Visualization of differentially expressed genes (DEGs) supporting the detailed identified domains. (**A**) Manual annotations and comparison of spatial domains identified by SpaConTDS, STAGATE, ConST, GraphST, stLearn, SpaGCN, scanpy, Louvain, ConGI, IRIS, Miso, and MorphLink on IDC dataset with DB index. (**B**) Manual annotations and comparison of spatial domains identified by SpaConTDS, STAGATE, ConST, GraphST, stLearn, SpaGCN, scanpy, Louvain, ConGI, IRIS, Miso, and MorphLink on human breast cancer dataset with ARI and NMI. (**C**)Violin plots of expression of DEGs (SERHL2, CRISP3, DUSP23, EIF3H, UBE2S, IGFBP5, NUPR1, COX7C) in subcluster 2 and 15 versus other clusters. (**D**)Top: raw gene expression patterns of DEGs. Bottom: denoised gene expression patterns of DEGs.
(PDF)

S6 Fig Batch effect correction ability of SpaConTDS. (**A**) UMAP plots after batch effect correction on 4 DLPFC slices. (**B**) UMAP plots after batch effect correction on human placental bed dataset slices. (**C**) Spatial domains identified by SpaConTDS on human placental bed dataset.
(PDF)

S7 Fig Comparison of spatial clustering and interface domains identified by SpaConTDS, STAGATE, ConST, GraphST, stLearn, SpaGCN, scanpy, Louvain, ConGI, IRIS, Miso, and MorphLink on slices A and B with DB index. (PDF)

S8 Fig Comparison of spatial domains identified by SpaConTDS, Miso, and MorphLink on the human tonsil dataset comprising three modalities with DB index. (PDF)
