# Supplementary material for: SpaConTDS: A multimodal contrastive learning framework for identifying spatial domains by applying tuple disturbing strategy
Source: PLoS Comput Biol. 2026 Jan 29;22(1):e1013893. doi: 10.1371/journal.pcbi.1013893 (PMC12854462; doi:10.1371/journal.pcbi.1013893)
Supplement: S1 Text — (PDF) [file pcbi.1013893.s001.pdf]

SpaConTDS: A multimodal contrastive learning framework for identifying spatial domains and integration

Supplementary Note

1 Data Summary

For spatial clustering, we applied SpaConTDS to 8 ST datasets generated from different platforms, including 10x Visium, ST, and 10x Xenium, they are listed as follows:

- The **HER2-positive Breast Cancer (HER2+)** dataset from ST platform consists of 8 slices[1], which was downloaded from (https://github.com/almaan/HER2st/). Since the number of spots in the C1 slice is fewer than 200, the experiment was not conducted on this slice. The number of spots in the other 7 slices ranges from 295 to 691, and each slice contains 3-6 regions. This dataset was used to validate that SpaConTDS facilitates accurate spatial domain identification and downstream tasks, and its performance was superior to other benchmark algorithms. The corresponding results were shown in Figs 2A-C,2E,7E,S1, and S2.

- The **Dorsolateral Prefrontal Cortex (DLPFC)** dataset includes 12 tissue slices acquired from 10x Visium platform[2], which was downloaded from (http://research.libd.org/spatialLIBD/). Each slice contains five or seven regions, namely the layers 1-6 and white matter. The number of spots varies from 3498 to 4789, with 33,538 genes captured. This dataset was used to validate that SpaConTDS facilitates accurate spatial domain identification and downstream tasks, even when the H&E images contain less information. The corresponding results were shown in Figs 2D-H,S3, and S4.

- The **Human Breast Cancer (HBC-10x)** dataset was also generated from 10x Visium platform, it contains 3,798 spots, and was manually annotated to 20 regions[3]. This dataset was used to study the tumor microenvironment in human breast cancer, and it revealed that SpaConTDS identified spatial domains showed superior concordance with pathological annotations. The corresponding results were shown in Figs 3A-C, and S5.

- The **Invasive Ductal Carcinoma (IDC)** dataset was generated from 10x Visium platform, it contains 4727 spots and 36,601 genes[4] without manual annotation. IDC dataset was downloaded from (https://support.10xgenomics.com/spatial-gene-expression/datasets), and was used to study the tumor microenvironment in invasive ductal carcinoma, it revealed that SpaConTDS effectively extracts biologically meaningful features specific to tumor regions, enabling precise characterization of the tumor microenvironment. The corresponding results were shown in Figs 3D-F, and S5.

- The **Zebrafish Melanoma** dataset was generated from 10x Visium platform, it contains 2 tissue slices, A and B[5], both capture 2179 spots and 32,268 genes without manual annotation. It was downloaded from (https://zenodo.org/records/10437391). This dataset was used to test the performance of SpaConTDS on resolving fine-scale regional distinctions at tumor margins, and it confirmed that its exceptional ability to delineate subtle tumor-stroma boundaries at cellular resolution. The corresponding results were shown in Figs 6A-C and S7.

- The **Human Placental Bed** dataset was generated from 10x Visium platform, it includes 3 tissue slices, Slice 64, Slice 65 and Slice 67[6]. The number of spots varied from 3568 to 4186, with 33,538 genes captured. It can be downloaded from (https://zenodo.org/records/10437391). This dataset was used to test

the performance of SpaConTDS on multiple sample integration and partial overlapping slices integration. The corresponding results were shown in Figs 4B,E and S6B,C.

- The **Human Tonsil** dataset was generated from Spatial CITE-seq platform[7], and was downloaded from (https://www.10xgenomics. com/datasets/gene-protein-expression-library-of-human-tonsilcytassist-ffpe-2-standard). This dataset comprises 4,194 spots, 18,060 genes, and 35 proteins, and was used to test whether SpaConTDS can be extended to multi-modalities. The corresponding results were shown in S8 Fig.

- Another **Human Breast Cancer (HBC-Xenium)** dataset was generated from 10x Xenium platform, containing 167,780 spots and 313 genes[8]. It can be downloaded from (https://www.10xgenomics.com/products/xenium in-situ/preview-dataset-human-breast/). This dataset was used to test the performance of SpaConTDS on high-resolution data, and the computational experiment revealed that it exhibited robustness and scalability in HBC-Xenium dataset and was superior to other benchmark algorithms. The corresponding results were shown in Fig 5.

Information for each slice or section in the above datasets can be find in S1 Table.

2 State-of-the-Art Algorithms

To showcase the effectiveness of SpaConTDS, we compared SpaConTDS with eleven state-of-the-art methods, the nonspatial method Louvain[9], unimodal spatial methods ConST[10], STAGATE[11], GraphST[12], Scanpy[13], and multimodal spatial methods stLearn[14], SpaGCN[15], ConGI[16], IRIS[17], MISO[18] and MorphLink[19]. This section presents a comparative overview of these advanced algorithms, highlighting their unique characteristics and contributions.

Louvain. Louvain is a well-established community detection algorithm used for clustering, which operates by optimizing modularity to partition spots into communities based on gene expression information. Here, we directly used the Python package 'community_louvain', utilizing the default parameters provided within it.

conST. conST is a contrastive learning-based method that uses three levels of tasks to combine gene expression, spatial information, and morphology for ST data analysis. We applied conST for spatial clustering with default parameter settings. Specifically, the number of neighbors was set to 20 when constructing the neighborhood graph, the training epoch and learning rate were set to 200 and 0.01, respectively.

STAGATE. STAGATE utilizes a graph attention mechanism combining an auto-encoder to learn representation by modeling both gene expression and spatial location information. This allows STAGATE to focus on the most relevant spatial features, improving domain identification. We ran STAGATE for spatial clustering and ST data integration. All experiments were implemented using the recommended parameters in the guidelines on the STAGATE GitHub repository (https://stagate.readthedocs.io/en/latest/index.html).

GraphST. GraphST combines graph neural networks with self-supervised contrastive learning to extract informative and discriminative spot representations from ST data, by minimizing the embedding distance between spatially adjacent spots and vice versa. We used GraphST with the default parameters for spatial clustering and ST data integration, according to the tutorial on the GraphST GitHub repository (https://deepst-tutorials.readthedocs.io/en/latest/).

Scanpy. Scanpy is a widely used tool for single-cell transcriptomics data analysis, known for its extensive functionalities including data preprocessing, dimensionality reduction, and clustering methods. Spatial clustering and ST data integration were performed using Scanpy's default parameters, where employing the Leiden method for clustering. Since directly specifying the cluster number in Leiden is not supported, we used binary search to dynamically adjust the resolution to achieve the desired number of clusters.

SpaGCN. SpaGCN integrates gene expression, spatial location, and histology image features using graph convolutional networks (GCNs). SpaGCN helps bridge the gap between gene expression and tissue structure, by leveraging the relationships between spots' spatial positions and histology image features, which enhances spatial domain identification. We applied SpaGCN to spatial clustering with the default parameter settings in the tutorial. In particular, the parameter 'histology' was set to 'True'. The learning rate and max training epoch were set to 0.05 and 200, respectively.

stLearn. stLearn normalizes gene expression data before analysis by incorporating spatial information and morphological features from histology images, that provides a flexible and powerful approach for understanding biological specimens, allowing for better clustering of spatial domains. We applied stLearn for spatial clustering

with default parameter settings in the the guideline. Specifically, the number of top components retained in PCA was set to 15, when reducing the dimension of features.

ConGI. ConGI adopts multimodal contrastive learning to learn a joint representation of gene expression and histology images at the spot level for spatial domain identification, by designing three contrastive loss functions within and between the two modalities to fully couple the information. We applied ConGI with the default parameters for spatial clustering analysis, as provided in the ConGI code.

IRIS. IRIS leverages single-cell RNA sequencing data for reference-informed detection of spatial domains in SRT studies, integrating multiple slices while explicitly considering correlations within and across slices to achieve high accuracy and efficiency. We used IRIS in 'IRISfree' mode with the default parameters for spatial clustering and ST data integration, following the tutorial on the IRIS GitHub repository (`https://yingma0107.github.io/IRIS/documentation/04_IRIS_Example.html`).

MISO. MISO integrates multiple spatial omics modalities by generating modality-specific embeddings and their interaction features via outer product, then applies quality-based feature filtration and k-means clustering to identify spatially distinct domains. We employed MISO with the default parameters for spatial clustering analysison both ST data and histological images, as specified in the MISO code.

MorphLink. MorphLink introduces a curve-based pattern similarity index (CPSI) to systematically identify and interpretively link tissue morphological features with molecular dynamics in spatial omics data by quantifying their local and global spatial pattern similarities, offering scalability and robustness for integrative analysis across modalities. We performed spatial clustering analysis on both ST data and histological images using MorphLink with default parameters, based on the MorphLink code.

3 Implementation Details

The histological image encoder consists of a pre-trained Hist2ST convmixer, followed by a learnable MLP[20] that uses ReLU as its activation function. The fully connected head of the image encoder projects the features into a latent space with dimensions [1024, 128]. The fusion module is implemented as an MLP with dimensions [256, 128], which consists solely of a linear projection layer, without any nonlinear activation functions. The gene encoder is a 2-layer GraphSAGE[21], using GELU activation, while the decoder is a 2-layer GCN[22] with Parametric ReLU (PReLU) activation.

The model is configured with different parameters for various datasets. For the 10x Visium[23] and ST data [24], the gene encoder uses dimensions [3000, 512, 128], while the decoder uses dimensions [128, 512, 3000].For the 10x Xenium data [25], which has a larger number of spots but a smaller gene dimension, the dimensions are set to [300, 200, 128] for the gene encoder and [128, 200, 300] for the decoder.

In gene modality data augmentation, noise was added with a mask probability 'gene_mask_pct' of 0.1 and a noise standard deviation 'gene_sigma_noise' of 1. For the image modality, data augmentation included random horizontal flip, vertical flip, and Gaussian blur, each applied with a probability of 0.3, where the Gaussian blur used a kernel size of 3x3, with a blur degree ranging from 0.1 to 2.0.

The initial perturbation probability $\boldsymbol{\alpha}$ for each modality is set to $[\frac{1}{3}, \frac{1}{3}, \frac{1}{3}]$, with the initial contribution factor k_{emb} for the gene modality set to 1.0 in datasets like DLPFC with low image information, and 0.5 in other datasets. Both hyperparameters are updated via reinforcement learning with coefficients set to 1. The reinforcement learning section sampled 'clone' times from the distribution, and each sample underwent 'internal' iterations. These two parameters control the number of sub-iterations in each epoch, with a total of 'clone $\times$ internal' sub-loops per epoch. When the number of spots is less than 1000, 'clone' and 'internal' are set to 5 and 6, respectively. To improve efficiency, when the number of spots is greater than 1000, these are set to 3 and 3.

The number of clusters for the validation set and pseudo-clusters are set to the number of actual categories, with a temperature hyperparameter τ set to 0.07. Additionally, we optimized SpaConTDS using Adam[26] with a learning rate of 0.01 and a batch size of 128. The loss function includes three components, with the hyperparameters $\gamma 1$ and $\gamma 2$ used to balancing these components, both set to 1.

For the baseline methods compared in this study, we primarily used their default parameters or those recommended in their official documentation, with specific adjustments as follows. ConST was configured with a neighborhood size of 20, 200 training epochs, and a learning rate of 0.01. SpaGCN was set with 'histology=True', a learning rate of 0.05, and 200 training epochs. stLearn retained the top 15 principal components during dimensionality reduction. Notably, since Scanpy (specifically, Leiden) cannot directly specify the number of clusters, it dynamically adjusted the resolution via binary search.

4 Extending to Multi-modalities

Our framework can be naturally extended to multi-modal settings. In the multi-modal encoder–decoder module, each omics modality adopts the same encoder design as the spatial transcriptomics encoder. Specifically, GraphSAGE is employed as the backbone, with layer dimensions adjusted according to the input feature dimensionality of the corresponding modality. As an illustrative example, we consider the Human Tonsil spatial CITE-seq dataset, in which the spatial protein modality has a feature dimension of 35. Accordingly, the spatial protein encoder is configured with layer sizes [35, 64, 128]. Here 35 is the dimension for input layer, 64 is the dimension for hidden layer and 128 is the dimension for output layer. Then the corresponding decoder is symmetrically defined with layer sizes [128, 64, 35]. For image-based modalities, the encoder architecture is identical to that used for histological images. It consists of a pre-trained Hist2ST ConvMixer for feature extraction, followed by a trainable multilayer perceptron (MLP) for representation learning. Finally, given k input modalities, the modality fusion module is implemented as an MLP with layer size [128 $\times k$, 128], which integrates modality-specific embeddings into a unified latent representation.

In the contrastive learning module, the construction of positive samples follows the same strategy as in the two-modality setting. Specifically, for the omics modality, the augmentation strategy is identical to that used for the ST modality, while for the image modality, the augmentation strategy is consistent with that of the histological modality. The construction of negative samples also follows a strategy similar to that in the two-modality setting. Let $\mathbf{h_i} = (\mathbf{v}_{i1}, \mathbf{v}_{i2}, \cdots, \mathbf{v}_{ik})$ denote a k-tuple anchor, where $\mathbf{v}_{im}, m \in \{1, 2, \cdots, k\}$ represents the m-th tuple components. Based on this formulation, we define k types of negative samples, denoted by $\mathbf{h_{ij}^{l-}}$, in which l tuple components of $\mathbf{h}_i$ are randomly replaced by the corresponding components from $\mathbf{h}_j$. The final negative sample corresponding to $\mathbf{h_i}$ is then constructed as a weighted combination of those variants,

$$\mathbf{h}_{ij}^{-} = \alpha_0 \mathbf{h}_i + \sum_{l} \alpha_l \mathbf{h}_{ij}^{l-}.$$

The form of the contrastive loss remains the same as that used in the manuscript. The hyperparameters k_{emb} and $\alpha = \{\alpha_0, \alpha_1, \cdots, \alpha_k\}$ are updated in the similar manner as described in Algorithm 1, with initial values set to $k_{emd} = 1/k$ and $\alpha_i = 1/k, i = 1, 2, \cdots, k$. Taking three modalities as an example, in contrastive learning, the initialized negative-sample perturbation probabilities remain $\alpha = [1/3, 1/3, 1/3]$, meaning perturb any 1 random modality with probability $\alpha[0] = 1/3$, perturb any 2 random modalities with probability $\alpha[1] = 1/3$, and perturb all 3 modalities with probability $\alpha[2] = 1/3$. In fusion stage, each modality is assigned an equal initial contribution factor k_{emb} is $[1/3, 1/3, 1/3]$. The fused embedding is then computed as:

$$\mathbf{h}_{\text{fused}} = \sum_{i=1}^{3} k_{\text{emb}}[i-1] \cdot \mathbf{h}_{\text{modality}_i}, \tag{1}$$

where $\mathbf{h}_{\text{modality}_1}$, $\mathbf{h}_{\text{modality}_1}$, and $\mathbf{h}_{\text{modality}_1}$ denote the embedding produced by the histological image, spatial transcriptomics, and spatial protein encoders, respectively. And the updating rules for α and k_{emb} follows

$$\mathcal{R}(\alpha_i) = \frac{\text{ARI}(l_{\alpha_i}^{img}, l_{\alpha_i}^{ST}) + \text{ARI}(l_{\alpha_i}^{img}, l_{\alpha_i}^{pro}) + \text{ARI}(l_{\alpha_i}^{pro}, l_{\alpha_i}^{ST}) - (\text{ARI}(l_{\mu_t}^{img}, l_{\mu_t}^{ST}) + \text{ARI}(l_{\mu_t}^{img}, l_{\mu_t}^{pro}) + \text{ARI}(l_{\mu_t}^{pro}, l_{\mu_t}^{ST}))}{\text{ARI}(l_{\mu_t}^{img}, l_{\mu_t}^{ST}) + \text{ARI}(l_{\mu_t}^{img}, l_{\mu_t}^{pro}) + \text{ARI}(l_{\mu_t}^{pro}, l_{\mu_t}^{ST})} \tag{2}$$

5 Evaluation Criteria

To evaluate the clustering performance, we used three metrics: adjusted Rand index (ARI) [27], Normalized Mutual Information (NMI)[28], and Davies-Bouldin index (DB)[29].

ARI measures the similarity between the ground truth and the cluster assignment predicted by the algorithm, while correcting for the chance grouping of elements:

$$ARI = \frac{\sum_i \sum_j \binom{n_{ij}}{2} - \left[\sum_i \binom{a_i}{2}\binom{b_j}{2}\right] / \binom{n}{2}}{\left[\sum_i \binom{a_i}{2} + \binom{b_j}{2}\right] - \left[\sum_i \binom{a_i}{2}\binom{b_j}{2}\right] / \binom{n}{2}}, \tag{3}$$

where n is the number of spots, a_i is the number of spots that belong to annotation cluster X_i, b_j is the number of spots assigned to predict cluster Y_j, and n_{ij} is the number of spots that belong to annotation cluster X_i but are assigned to predicted cluster Y_j.

Although ARI effectively evaluates the agreement of clustering results while adjusting for the influence of randomness, it primarily focuses on the pairing relationships between sample pairs and may not fully reflect the information sharing and structural integrity between clusters. In contrast, Normalized Mutual Information (NMI) quantifies the degree of information sharing between the clustering results, effectively eliminating the effects of differing cluster counts and data distributions, thereby providing a more nuanced assessment of clustering performance. Essentially, NMI measures the amount of shared information between the ground truth and the predicted cluster, and normalizes it to account for the expected mutual information under random chance:

$$NMI = \frac{\sum_{u \in U} \sum_{v \in V} p(u,v) \log \frac{p(u,v)}{p(u)p(v)}}{\sqrt{H(U)H(V)}} \tag{4}$$

where

$$H(X) = -\sum_{x \in X} p(x) \log p(x). \tag{5}$$

$p(u,v)$ is he joint probability of a sample belonging to class u and class v, $p(u)$ and $p(v)$ are the marginal probability of class u and v, respectivly. $p(x)$ is the probability of class x. ARI and NMI both range from 0 to 1, the higher score means the better cluster assignment.

When the ground truth was not available, we used DB to evaluate the sample distribution in the feature space. DB measures the compactness within a cluster and the distance between clusters, quantifies how well-separated and internally cohesive the clusters are:

$$DB = \frac{1}{k} \sum_{i=1}^{k} \max_{j \neq i} \left(\frac{\sigma_i + \sigma_j}{d(c_i, c_j)} \right), \tag{6}$$

where k is the number of clusters, c_i is the centroid of C_i, $d(c_i, c_j)$ is the Euclidean distance between cluster centroids c_i and c_j, and σ_i is the mean distance between spots that belong to cluster C_i and centroid c_i. $DB \in [0, +\infty)$, the smaller the scores, the better the clustering performance.

6 Proof Of TupleInfoNCE

From an information-theoretic perspective, the TupleInfoNCE[30] framework demonstrates that under the setting of constructing negative samples through the tuple perturbation strategy, optimizing the InfoNCE loss function is equivalent to maximizing the lower bound of the following mutual information:

$$I(t_{2,i}, t_{1,i}) + \sum_{k=1}^{K} \alpha_k I(u_{i,k}; \overline{u}_{i,k}) \tag{7}$$

In this context, $I(t_{2,i}, t_{1,i})$ refers to the mutual information between the original view and the augmented view, while $I(v_{i,k}; \overline{v}_{i,k})$ signifies the mutual information between the k-th modality and the other modalities. Compare to traditional methods, the tuple perturbation strategy adds an additional term $I(v_{i,k}; \overline{v}_{i,k})$, representing the mutual information between modalities to the objective function being optimized.

On one hand, when modality k is a weak modality, maximizing $I(v_{i,k}; \overline{v}_{i,k})$ can alleviate the issue of the weak modality being overlooked to some extent. On the other hand, maximizing the mutual information $I(v_{i,k}; \overline{v}_{i,k})$ between modalities encourages the model to capture the interactions between modalities during the modality fusion process, rather than treating the modalities as independent. Here we provide the complete and detailed proof of TupleInfoNCE from an information theory perspective, that minimizing L_{NCE} is equivalent to maximizing the lower bound of equation(5).

Assume that each multimodal sample t contains K modalities $\{v_1, \cdots, v_K\}$. $t_{1,i}$ represents the original view of the i-th multimodal sample, $t_{2,i}$ represents the augmented view of the i-th multimodal sample, and $t_{2,j \neq i}$ represents the j-th negative sample constructed from the i-th multimodal sample. For each anchor $t_{1,i}$, construct 1 positive sample and $N-1$ negative samples. The anchor sample $t_{1,i}$, the augmented sample $t_{2,i}$, and the negative samples $t_{2,j \neq i}$ are all random variables, following the following distributions:

$$t_{1,j} \sim p_1(t) = p_1(v_k, \overline{v}_k); t_{2,i} \sim p_2(t_{2,i}|t_{1,i}), t_{2,j \neq i} \sim q_\alpha(t_{2,j \neq i}) \tag{8}$$

According to the tuple perturbation strategy, let the negative sample $t_{2,j \neq i}$ follow the mixture distribution $q_\alpha(t_{2,j \neq i})$. a_0 is the probability of all K modalities are replaced by the corresponding modalities from the j-th

sample. the α_k denotes the probability that only the k-th modality is replaced by the j-th sample, while the remaining modalities are the i-th sample.

$$q_\alpha(t_{2,j\neq i}) = q_\alpha(v_{j,k} \cdot \overline{v}_{j,k}) = a_0 p_1(t_{2,j\neq i}) + \sum_{k=1}^{K} \alpha_k p_1(v_{j,k}) p_1(\overline{v}_{j,k}) \tag{9}$$

The InfoNCE loss function is:

$$L_{NCE} = -\mathbb{E}_{(t_{1,i},t_{2,j},t_{2,j\neq i})}[\log \frac{f(t_{1,i},t_{2,i})}{\sum_{j=1}^{N} f(t_{1,i},t_{2,j})}] \tag{10}$$

where f is a scoring function used to measure the similarity between $t_{1,i}$ and $t_{2,j}$.

Proof: First, equation (8) can be viewed as a cross-entropy loss function for an N-class classification task, where the probability of $t_{2,i}$ being classified as class i is $\frac{f(t_{1,i},t_{2,i})}{\sum_{j=1}^{N} f(t_{1,i},t_{2,j})}$. The optimization goal is to maximize the probability of $t_{2,i}$ being predicted as the positive sample. Therefore, L_{NCE} takes the minimum value when $t_{2,i}$ has the highest probability of being predicted as the positive sample, at which point $t_{2,i}$ comes from the positive sample distribution $p_2(t_{2,i}|t_{1,i})$, and $t_{2,j\neq i}$ comes from the negative sample distribution $q_\alpha(t_{2,j\neq i})$. Assuming $t_{2,i}$ and $t_{2,j\neq i}$ are independent, we have:

$$\frac{f(t_{1,i},t_{2,i})}{\sum_{j=1}^{N} f(t_{1,i},t_{2,j})} = \frac{p_2(t_{2,i}|t_{1,i}) \prod_{j\neq i} q_\alpha(t_{2,j})}{\sum_{j=1}^{N} p_2(t_{2,j}|t_{1,i}) \prod_{j\neq i} q_\alpha(t_{2,j})} \tag{11}$$

Dividing both the numerator and the denominator of equation (9) by $\prod_{j=1}^{N} q_\alpha(t_{2,j})$, we get:

$$\frac{p_2(t_{2,i}|t_{1,i})/q_\alpha(t_{2,i})}{\sum_{j=1}^{N} p_2(t_{2,j}|t_{1,i})/q_\alpha(t_{2,j})} \tag{12}$$

From equations (9) and (10), we get $f(t_{1,i},t_{2,i}) = \frac{p_2(t_{2,i}|t_{1,i})}{q_\alpha(t_{2,i})}$, substitute it into equation (8), we obtain:

$$\begin{aligned}
L_{NCE}^{\text{OPT}} &= -\mathbb{E}_{(t_{1,i},t_{2,i},t_{2,j},j\neq i)}[\log \frac{\frac{p_2(t_{2,i}|t_{1,i})}{q_\alpha(t_{2,i})}}{\sum_{j=1}^{N} \frac{p_2(t_{2,j}|t_{1,i})}{q_\alpha(t_{2,j})}}] \\
&= -\mathbb{E}_{(t_{1,i},t_{2,i},t_{2,j},j\neq i)}[\log \frac{\frac{p_2(t_{2,i}|t_{1,i})}{q_\alpha(t_{2,i})} + \sum_{j\neq i} \frac{p_2(t_{2,j}|t_{1,i})}{q_\alpha(t_{2,j})}}{q_\alpha(t_{2,i})}] \\
&= \mathbb{E}_{(t_{1,i},t_{2,i},t_{2,j},j\neq i)}[\log \frac{\frac{p_2(t_{2,i}|t_{1,i})}{q_\alpha(t_{2,i})} + \sum_{j\neq i} \frac{p_2(t_{2,j}|t_{1,i})}{q_\alpha(t_{2,j})}}{\frac{p_2(t_{2,i}|t_{1,i})}{q_\alpha(t_{2,i})}}] \\
&= \mathbb{E}_{(t_{1,i},t_{2,i},t_{2,j},j\neq i)}[\log(1 + \frac{\sum_{j\neq i} \frac{p_2(t_{2,j}|t_{1,i})}{q_\alpha(t_{2,j})}}{\frac{p_2(t_{2,i}|t_{1,i})}{q_\alpha(t_{2,i})}})] \\
&= \mathbb{E}_{(t_{1,i},t_{2,i},t_{2,j},j\neq i)}[\log(1 + \frac{q_\alpha(t_{2,i})}{p_2(t_{2,i}|t_{1,i})} \sum_{j\neq i} \frac{p_2(t_{2,j}|t_{1,i})}{q_\alpha(t_{2,j})})]
\end{aligned} \tag{13}$$

By the law of large numbers, $\sum_{j\neq i} \frac{p_2(t_{2,j}|t_{1,i})}{q_\alpha(t_{2,j})} \approx (N-1)\mathbb{E}_{t_{2,j}}[\frac{p_2(t_{2,j}|t_{1,i})}{q_\alpha(t_{2,j})}]$, then

$$\begin{aligned}
L_{NCE}^{\text{OPT}} &= \mathbb{E}_{(t_{1,i},t_{2,i},t_{2,j},j\neq i)}[\log(1 + \frac{q_\alpha(t_{2,i})}{p_2(t_{2,i}|t_{1,i})} \sum_{j\neq i} \frac{p_2(t_{2,j}|t_{1,i})}{q_\alpha(t_{2,j})})] \\
&\approx \mathbb{E}_{(t_{1,i},t_{2,i},t_{2,j},j\neq i)}[\log(1 + \frac{q_\alpha(t_{2,i})}{p_2(t_{2,i}|t_{1,i})}(N-1)\mathbb{E}_{t_{2,j}}[\frac{p_2(t_{2,j}|t_{1,i})}{q_\alpha(t_{2,j})}])]
\end{aligned} \tag{14}$$

where,

$$\begin{aligned}
\mathbb{E}_{t_{2,j}}\left[\frac{p_2(t_{2,j}|t_{1,i})}{q_\alpha(t_{2,j})}\right] &= \int p(t_{2,j}) \frac{p_2(t_{2,j}|t_{1,i})}{q_\alpha(t_{2,j})}\, dt_{2,j} \\
&= \int p_2(t_{2,j}|t_{1,i}) \frac{p(t_{2,j})}{q_\alpha(t_{2,j})}\, dt_{2,j} \\
&= \mathbb{E}_{t_{2,j}\sim p_2(t_{2,j}|t_{1,i})}\left[\frac{p(t_{2,j})}{q_\alpha(t_{2,j})}\right]
\end{aligned} \tag{15}$$

In equation (13), $p(t_{2,j})$ represents the true distribution of $t_{2,j}$, and $q_\alpha(t_{2,j})$ represents the distribution of $t_{2,j}$ as a negative sample. When L_{NCE} takes the minimum value, $p(t_{2,j}) = q_\alpha(t_{2,j})$, then $\mathbb{E}_{t_{2,j}}[\frac{p_2(t_{2,j}|t_{1,i})}{q_\alpha(t_{2,j})}] = \mathbb{E}_{t_{2,j}\sim p_2(t_{2,j}|t_{1,i})}[\frac{p(t_{2,j})}{q_\alpha(t_{2,j})}] = 1$. Substituting into equation (12), we get:

$$
\begin{aligned}
L_{NCE}^{\text{OPT}} &\approx \mathbb{E}_{(t_{1,i},t_{2,i},t_{2,j},j\neq i)}[\log(1+\frac{q_\alpha(t_{2,i})}{p_2(t_{2,i}|t_{1,i})}(N-1))] \\
&= \mathbb{E}_{(t_{1,i},t_{2,i},t_{2,j},j\neq i)}[\log(\frac{q_\alpha(t_{2,i})}{p_2(t_{2,i}|t_{1,i})}N + (1-\frac{q_\alpha(t_{2,i})}{p_2(t_{2,i}|t_{1,i})}))]
\end{aligned} \tag{16}
$$

When L_{NCE} takes the minimum value, $t_{2,i}$ is a positive sample, then the probability that $t_{2,i}$ is a negative sample is less than or equal to the probability that $t_{2,i}$ is a positive sample, i.e., $q_\alpha(t_{2,i}) \leq p_2(t_{2,i}|t_{1,i})$, therefore,

$$
\begin{aligned}
L_{NCE}^{\text{OPT}} &\approx \mathbb{E}_{(t_{1,i},t_{2,i},t_{2,j},j\neq i)}[\log(\frac{q_\alpha(t_{2,i})}{p_2(t_{2,i}|t_{1,i})}N + (1-\frac{q_\alpha(t_{2,i})}{p_2(t_{2,i}|t_{1,i})}))] \\
&\geq \mathbb{E}_{(t_{1,i},t_{2,i},t_{2,j},j\neq i)}[\log(\frac{q_\alpha(t_{2,i})}{p_2(t_{2,i}|t_{1,i})}N)] \\
&= \mathbb{E}_{(t_{1,i},t_{2,i},t_{2,j},j\neq i)}[\log(\frac{a_0 p_1(t_{2,i}) + \sum_{k=1}^{K} a_k p_2(v_{i,k}) p_3(\bar{t}_{i,k})}{p_2(t_{2,i}|t_{1,i})}N)]
\end{aligned} \tag{17}
$$

In equation (15), $a_0 p_1(t_{2,i}) + \sum_{k=1}^{K} a_k p_2(v_{i,k}) p_3(\bar{t}_{i,k})$ can be regarded as the expectation of a discrete random variable x, and the distribution of x is as follows:

$$
\begin{cases}
P(x = p_1(t_{2,i})) = a_0 \\
P(x = p_2(v_{i,k}) p_3(\bar{t}_{i,k})) = a_k, \quad k = 1, \cdots, K
\end{cases} \tag{18}
$$

By Jensen's inequality $\log \mathbb{E}_x[f(x)] \geq \mathbb{E}_x[\log(f(x))]$, we know:

$$
\begin{aligned}
\log(a_0 p_1(t_{2,i}) + \sum_{k=1}^{K} a_k p_2(v_{i,k}) p_3(\bar{t}_{i,k})) &\geq a_0 \log(p_1(t_{2,i})) + \sum_{k=1}^{K} a_k \log(p_2(v_{i,k}) p_3(\bar{t}_{i,k})) \\
&= \log[p_1(t_{2,i})^{a_0} \prod_{k=1}^{K} (p_2(v_{i,k}) p_3(\bar{t}_{i,k}))^{a_k}]
\end{aligned} \tag{19}
$$

Substitute equation (17) into equation (15),

$$
\begin{aligned}
L_{NCE}^{\text{OPT}} &\geq \mathbb{E}_{(t_{1,i},t_{2,i},t_{2,j},j\neq i)}[\log(\frac{a_0 p_1(t_{2,i}) + \sum_{k=1}^{K} a_k p_2(v_{i,k}) p_3(\bar{t}_{i,k})}{p_2(t_{2,i}|t_{1,i})}N)] \\
&\geq \mathbb{E}_{(t_{1,i},t_{2,i},t_{2,j},j\neq i)}[\log(\frac{p_1(t_{2,i})^{a_0} \prod_{k=1}^{K} (p_2(v_{i,k}) p_3(\bar{t}_{i,k}))^{a_k}}{p_2(t_{2,i}|t_{1,i})}N)]
\end{aligned} \tag{20}
$$

Since $a_0 + \sum_{k=1}^{K} a_k = 1$, we get:

$$
\begin{aligned}
L_{NCE}^{\text{OPT}} &\geq \mathbb{E}_{(t_{1,i},t_{2,i},t_{2,j},j\neq i)}[\log(N \cdot \frac{p_1(t_{2,i})^{a_0} \prod_{k=1}^{K} (p_2(v_{i,k}) p_3(\bar{t}_{i,k}))^{a_k}}{p_2(t_{2,i}|t_{1,i})})] \\
&= \mathbb{E}_{(t_{1,i},t_{2,i},t_{2,j},j\neq i)}[\log(N \cdot \frac{p_1(t_{2,i})}{p_2(t_{2,i}|t_{1,i})} \cdot p_1(t_{2,i})^{a_0 - 1} \cdot \prod_{k=1}^{K} (p_2(v_{i,k}) p_3(\bar{t}_{i,k}))^{a_k})] \\
&= \mathbb{E}_{(t_{1,i},t_{2,i},t_{2,j},j\neq i)}[\log(N \cdot \frac{p_1(t_{2,i})}{p_2(t_{2,i}|t_{1,i})} \cdot \frac{\prod_{k=1}^{K} (p_2(v_{i,k}) p_3(\bar{t}_{i,k}))^{a_k}}{p_1(t_{2,i})^{\sum_{k=1}^{K} a_k}})] \\
&= \log N + \mathbb{E}_{(t_{1,i},t_{2,i},t_{2,j},j\neq i)}[\log \frac{p_1(t_{2,i})}{p_2(t_{2,i}|t_{1,i})}] + \mathbb{E}_{(t_{1,i},t_{2,i},t_{2,j},j\neq i)}[\log \prod_{k=1}^{K} (\frac{p_2(v_{i,k}) p_3(\bar{t}_{i,k})}{p_1(t_{2,i})})^{a_k}] \\
&= \log N + \mathbb{E}_{(t_{1,i},t_{2,i},t_{2,j},j\neq i)}[\log \frac{p_1(t_{2,i})}{p_2(t_{2,i}|t_{1,i})}] + \sum_{k=1}^{K} a_k \mathbb{E}_{(t_{1,i},t_{2,i},t_{2,j},j\neq i)}[\log \frac{(p_2(v_{i,k}) p_3(\bar{t}_{i,k}))}{p_1(t_{2,i})}]
\end{aligned} \tag{21}
$$

By the following mutual information formula:

$$I(x;y) = \mathbb{E}_{(x,y)}[\log \frac{p(x,y)}{p(x)p(y)}] = \mathbb{E}_{(x,y)}[\log \frac{p(x,y)}{p(x)p(y)}] \tag{22}$$

We can get:

$$\begin{aligned} \mathbb{E}_{(t_{1,i},t_{2,i},t_{2,j},j\neq i)}[\log \frac{p_1(t_{2,i})}{p_2(t_{2,i}|t_{1,i})}] &= \mathbb{E}_{t_{2,i},t_{1,i}}[\log \frac{p_1(t_{2,i})}{p_2(t_{2,i}|t_{1,i})}] \\ &= -I(t_{2,i};t_{1,i}) \end{aligned} \tag{23}$$

We can also get:

$$\begin{aligned} &\sum_{k=1}^{K} a_k \mathbb{E}_{(t_{1,i},t_{2,i},t_{2,j},j\neq i)}[\log \frac{(p_2(v_{i,k})p_3(\bar{t}_{i,k}))}{p_1(t_{2,i})}] \\ &= -\sum_{k=1}^{K} a_k \mathbb{E}_{(t_{1,i},t_{2,i},t_{2,j},j\neq i)}[\log \frac{p_1(t_{2,i})}{(p_2(v_{i,k})p_3(\bar{t}_{i,k}))}] \\ &= -\sum_{k=1}^{K} a_k \mathbb{E}_{(t_{1,i},t_{2,i},t_{2,j},j\neq i)}[\log \frac{p_1(v_{i,k}\cdot \overline{v_{i,k}})}{p_2(v_{i,k})p_3(\bar{t}_{i,k})}] \\ &= -\sum_{k=1}^{K} a_k \mathbb{E}_{t_{2,j}}[\log \frac{p_1(v_{i,k}\cdot \overline{v_{i,k}})}{p_2(v_{i,k})p_3(\bar{t}_{i,k})}] \\ &= -\sum_{k=1}^{K} a_k \mathbb{E}_{(v_{i,k},\overline{v_{i,k}})}[\log \frac{p_1(v_{i,k}\cdot \overline{v_{i,k}})}{p_2(v_{i,k})p_3(\bar{v}_{i,k})}] \\ &= -\sum_{k=1}^{K} a_k I(v_{i,k};\overline{v_{i,k}}) \end{aligned} \tag{24}$$

Substitute equation (21) and equation (22) into equation (19):

$$\begin{aligned} & L_{NCE}^{\text{OPT}} \\ &\geq \log N + \mathbb{E}_{(t_{1,i},t_{2,i},t_{2,j},j\neq i)}[\log \frac{p_1(t_{2,i})}{p_2(t_{2,i}|t_{1,i})}] + \sum_{k=1}^{K} a_k \mathbb{E}_{(t_{1,i},t_{2,i},t_{2,j},j\neq i)}[\log \frac{(p_2(v_{i,k})p_3(\bar{t}_{i,k}))}{p_1(t_{2,i})}] \\ &= \log N - I(t_{2,i};t_{1,i}) - \sum_{k=1}^{K} a_k I(v_{i,k};\overline{v_{i,k}}) \end{aligned} \tag{25}$$

Thus,

$$I(t_{2,i};t_{1,i}) + \sum_{k=1}^{K} a_k I(v_{i,k};\overline{v_{i,k}}) \geq \log N - L_{NCE}^{\text{OPT}} \tag{26}$$

From equation (24), it can be deduced that minimizing L_{NCE} is equivalent to maximizing $I(t_{2,i};t_{1,i}) + \sum_{k=1}^{K} a_k I(v_{i,k};\bar{v}_{i,k})$.

References

[1] A. Andersson, L. Larsson, L. Stenbeck, F. Salmén, A. Ehinger, S. Z. Wu, G. Al-Eryani, D. Roden, A. Swarbrick, Å. Borg, et al., *Spatial deconvolution of HER2-positive breast cancer delineates tumor-associated cell type interactions*, Nature Communications, vol. 12, no. 1, pp. 6012, 2021.

[2] K. R. Maynard, L. Collado-Torres, L. M. Weber, C. Uytingco, B. K. Barry, S. R. Williams, J. L. Catallini, M. N. Tran, Z. Besich, M. Tippani, et al., *Transcriptome-scale spatial gene expression in the human dorsolateral prefrontal cortex*, Nature Neuroscience, vol. 24, no. 3, pp. 425–436, 2021.

[3] H. Xu, H. Fu, Y. Long, K. S. Ang, R. Sethi, K. Chong, M. Li, R. Uddamvathanak, H. K. Lee, J. Ling, et al., *Unsupervised spatially embedded deep representation of spatial transcriptomics*, Genome Medicine, vol. 16, no. 1, p. 12, 2024.

[4] E. Zhao, M. R. Stone, X. Ren, et al., Spatial transcriptomics at subspot resolution with BayesSpace. *Nat. Biotechnol.* **39**, 1375–1384 (2021).

[5] M. V. Hunter, R. Moncada, J. M. Weiss, I. Yanai, R. M. White, *Spatially resolved transcriptomics reveals the architecture of the tumor-microenvironment interface*, Nature Communications, vol. 12, no. 1, p. 6278, 2021.

[6] A. Arutyunyan, K. Roberts, K. Troulé, F. C. K. Wong, M. A. Sheridan, I. Kats, L. Garcia-Alonso, B. Velten, R. Hoo, E. R. Ruiz-Morales, et al., *Spatial multiomics map of trophoblast development in early pregnancy*, Nature, vol. 616, no. 7955, pp. 143–151, 2023.

[7] 10xGenomics.*https://www.10xgenomics.com/datasets/gene-protein-expression-library-of-human-tonsilcytassist-ffpe-2-standard*,2023.

[8] A. L. Ji, A. J. Rubin, K. Thrane, S. Jiang, D. L. Reynolds, R. M. Meyers, M. G. Guo, B. M. George, A. Mollbrink, J. Bergenstråhle, L. Larsson, Y. Bai, B. Zhu, A. Bhaduri, J. M. Meyers, X. Rovira-Clavé, S. T. Hollmig, S. Z. Aasi, G. P. Nolan, P. A. Khavari, Multimodal analysis of composition and spatial architecture in human squamous cell carcinoma. *Cell* **182**, 497–514 (2020) .

[9] V. A. Traag, L. Waltman, and N. J. Van Eck, From Louvain to Leiden: guaranteeing well-connected communities. *Sci Rep* **9**, 5233 (2019).

[10] Y. Zong, T. Yu, X. Wang, Y. Wang, Z. Hu, et al., conST: an interpretable multi-modal contrastive learning framework for spatial transcriptomics. Preprint at *bioRxiv* `https://doi.org/10.1101/2022.01.14.476408` (2022).

[11] K. Dong and S. Zhang, Deciphering spatial domains from spatially resolved transcriptomics with an adaptive graph attention auto-encoder. *Nat. Commun.* **13**, 1739 (2022) .

[12] Y. Long, et al., Spatially informed clustering, integration, and deconvolution of spatial transcriptomics with GraphST. *Nat. Commun.* **14**, 1155 (2023).

[13] F. A. Wolf, P. Angerer, and F. J. Theis, SCANPY: large-scale single-cell gene expression data analysis. *Genome biology* **19**, 1–5 (2018).

[14] D. Pham, X. Tan, X. Jun, et al., stLearn: integrating spatial location, tissue morphology and gene expression to find cell types, cell-cell interactions and spatial trajectories within undissociated tissues. *bioRxiv* (2020). `https://doi.org/10.1101/2020.05.31.125658v1`.

[15] H. Jian, X. Li, K. Coleman, et al., SpaGCN: integrating gene expression, spatial location and histology to identify spatial domains and spatially variable genes by graph convolutional network. *Nat. Methods* **18**, 1342–1351 (2021) .

[16] Y. Zeng et al., *Identifying spatial domain by adapting transcriptomics with histology through contrastive learning.* Briefings in Bioinformatics, 2023, 24(2): bbad048.

[17] Y. Ma and X. Zhou, *Accurate and efficient integrative reference-informed spatial domain detection for spatial transcriptomics.* Nature Methods, 2024, 21(7): 1231-1244.

[18] K. Coleman et al., *Resolving tissue complexity by multimodal spatial omics modeling with MISO.* Nature methods, 2025, 22(3): 530-538.

[19] J. Huang et al., *Bridging cell morphological behaviors and molecular dynamics in multi-modal spatial omics with MorphLink.* Nature Communications, 2025, 16(1): 5878.

[20] F. Rosenblatt, *Principles of Neurodynamics: Perceptrons and the Theory of Brain Mechanisms.* Washington, D.C.: Spartan Books, 1962.

[21] W. Hamilton, Z. Ying, and J. Leskovec, Inductive representation learning on large graphs. In *Advances in Neural Information Processing Systems*, vol. 30, 2017.

[22] T. N. Kipf and M. Welling, Semi-supervised classification with graph convolutional networks. In *Proceedings of the 5th International Conference on Learning Representations*, Toulon, France, 2017.

[23] A. L. Ji, A. J. Rubin, K. Thrane, S. Jiang, D. L. Reynolds, R. M. Meyers, M. G. Guo, B. M. George, A. Mollbrink, J. Bergenstråhle, L. Larsson, Y. Bai, B. Zhu, A. Bhaduri, J. M. Meyers, X. Rovira-Clavé, S. T. Hollmig, S. Z. Aasi, G. P. Nolan, P. A. Khavari, Multimodal analysis of composition and spatial architecture in human squamous cell carcinoma. *Cell* **182**, 497–514 (2020) .

[24] P. L. Ståhl, F. Salmén, S. Vickovic, et al., Visualization and analysis of gene expression in tissue sections by spatial transcriptomics. *Science* **353**, 78–82 (2016) .

[25] M. Salas, S. et al., Optimizing Xenium In Situ data utility by quality assessment and best practice analysis workflows. *bioRxiv* (2023). doi: `https://doi.org/10.1101/2023.02.13.528102`.

[26] D. P. Kingma and J. Ba, *Adam: A method for stochastic optimization.* arXiv preprint arXiv:1412.6980, 2014.

[27] D. Steinley, Properties of the Hubert-Arabie adjusted Rand index. *Psychological Methods*, **9**(3):386, 2004.

[28] A. F. McDaid, D. Greene, and N. Hurley, *Normalized mutual information to evaluate overlapping community finding algorithms.* arXiv preprint arXiv:1110.2515, 2011.

[29] D. L. Davies and D. W. Bouldin, *A cluster separation measure. IEEE Transactions on Pattern Analysis and Machine Intelligence*, Vol. 2, pp. 224–227, 2009.

[30] Y. Liu, Q. Fan, S. Zhang, H. Dong, T. Funkhouser, and L. Yi, *Contrastive multimodal fusion with TupleInfoNCE.* In *Proceedings of the IEEE/CVF International Conference on Computer Vision*, pages 754–763, 2021.
