# Supplementary material for: SpaConTDS: A multimodal contrastive learning framework for identifying spatial domains by applying tuple disturbing strategy
Source: PLoS Comput Biol. 2026 Jan 29;22(1):e1013893. doi: 10.1371/journal.pcbi.1013893 (PMC12854462; doi:10.1371/journal.pcbi.1013893)
Supplement: S1 Table — (PDF) [file pcbi.1013893.s002.pdf]

S1 Table. Description of all ST datasets used in SpaConTDS

Platform	Data set	Section	Spots	Genes	Related figures	Source
**ST**	**HER2+**	**A1**	**346**	**10262**	Fig 1B, Fig 6E, S1 Fig, S2 Fig	https://github.com/almaan/HER2st/
		**B1**	**295**	**9945**	Fig 1B, Fig 6E, S1 Fig, S2 Fig	
		**D1**	**306**	**12025**	Fig 1A,B,C, Fig 6E, S1 Fig, S2 Fig	
		**E1**	**587**	**8405**	Fig 1B, Fig 6E, S1 Fig, S2 Fig	
		**F1**	**691**	**11016**	Fig 1B, Fig 6E, S1 Fig, S2 Fig	
		**G2**	**467**	**10108**	Fig 1B, Fig 6E, S1 Fig, S2 Fig	
		**H1**	**613**	**9021**	Fig 1B, Fig 6E, S1 Fig, S2 Fig	
**10x Visium**	**DLPFC**	**151507**	**4226**	**15818**	Fig 1E, S3 Fig, S4 Fig	http://research.libd.org/spatialLIBD/
		**151508**	**4384**	**15353**	Fig 1E, S3 Fig, S4 Fig	
		**151509**	**4789**	**16033**	Fig 1E, S3 Fig, S4 Fig	
		**151510**	**4634**	**15820**	Fig 1E, S3 Fig, S4 Fig	
		**151669**	**3661**	**15971**	Fig 1E, S3 Fig, S4 Fig	
		**151670**	**3498**	**15701**	Fig 1E, S3 Fig, S4 Fig	
		**151671**	**4110**	**16355**	Fig 1D,E,F,G,H, S3 Fig, S4 Fig	
		**151672**	**4015**	**16058**	Fig 1E, S3 Fig, S4 Fig	
		**151673**	**3639**	**16578**	Fig 1E, Fig 3A,C,D, Fig 6A,B S3 Fig, S4 Fig, S6A Fig	
		**151674**	**3673**	**17332**	Fig 1E, Fig 3A,C,D, S3 Fig, S4 Fig, S6A Fig	
		**151675**	**3592**	**16062**	Fig 1E, Fig 3A,C,D, S3 Fig, S4 Fig, S6A Fig	
		**151676**	**3460**	**16097**	Fig 1E, Fig 3A,C,D, S3 Fig, S4 Fig, S6A Fig	
	**HBC-10x**		**3798**	**19690**	Fig 2A,B,C, S5B-D Fig	[1]
	**IDC**		**4727**	**36,601**	Fig 2D,E,F, Fig 6C,D, S5A Fig	https://support.10xgenomics.com/spatial-gene-datasets
	**Zebrafish Melanoma**	**slice A**	**2179**	**32,268**	Fig 5A-C, S7top Fig	https://zenodo.org/records/10437391

Platform	Data set	Section	Spots	Genes	Related figures	Source
		**slice B**	**2179**	**32,268**	Fig 5A,B, S7bottom Fig	https://zenodo.org/records/10437391
	**Human Placental Bed**	**slice 64**	**3568**	**33,538**	Fig 3B,E, S6B-C Fig	https://zenodo.org/records/10437391
		**slice 65**	**3855**	**33,538**	Fig 3B,E, S6B-C Fig	https://zenodo.org/records/10437391
		**slice 67**	**4186**	**33,538**	Fig 3B,E, S6B-C Fig	https://zenodo.org/records/10437391
**Spatial CITE-seq**	**Human Tonsil**		**4,194**	**18,060 35 prot**	S8 Fig	https://www.10xgenomics.com/datasets/gene-protein-expression-library-of-human-tonsilcytassist-ffpe-2-standard
**10x Xenium**	**HBC-Xenium**		**167,780**	**313**	Fig 4	https://www.10xgenomics.com/products/xeniumin-situ/preview-dataset-human-breast/

References

[1] H. Xu, H. Fu, Y. Long, K. S. Ang, R. Sethi, K. Chong, M. Li, R. Uddamvathanak, H. K. Lee, J. Ling, et al., *Unsupervised spatially embedded deep representation of spatial transcriptomics*, Genome Medicine, vol. 16, no. 1, p. 12, 2024.
