# Supplementary material for: SpaConTDS: A multimodal contrastive learning framework for identifying spatial domains by applying tuple disturbing strategy
Source: PLoS Comput Biol. 2026 Jan 29;22(1):e1013893. doi: 10.1371/journal.pcbi.1013893 (PMC12854462; doi:10.1371/journal.pcbi.1013893)
Supplement: S4 Fig — (PDF) [file pcbi.1013893.s006.pdf]

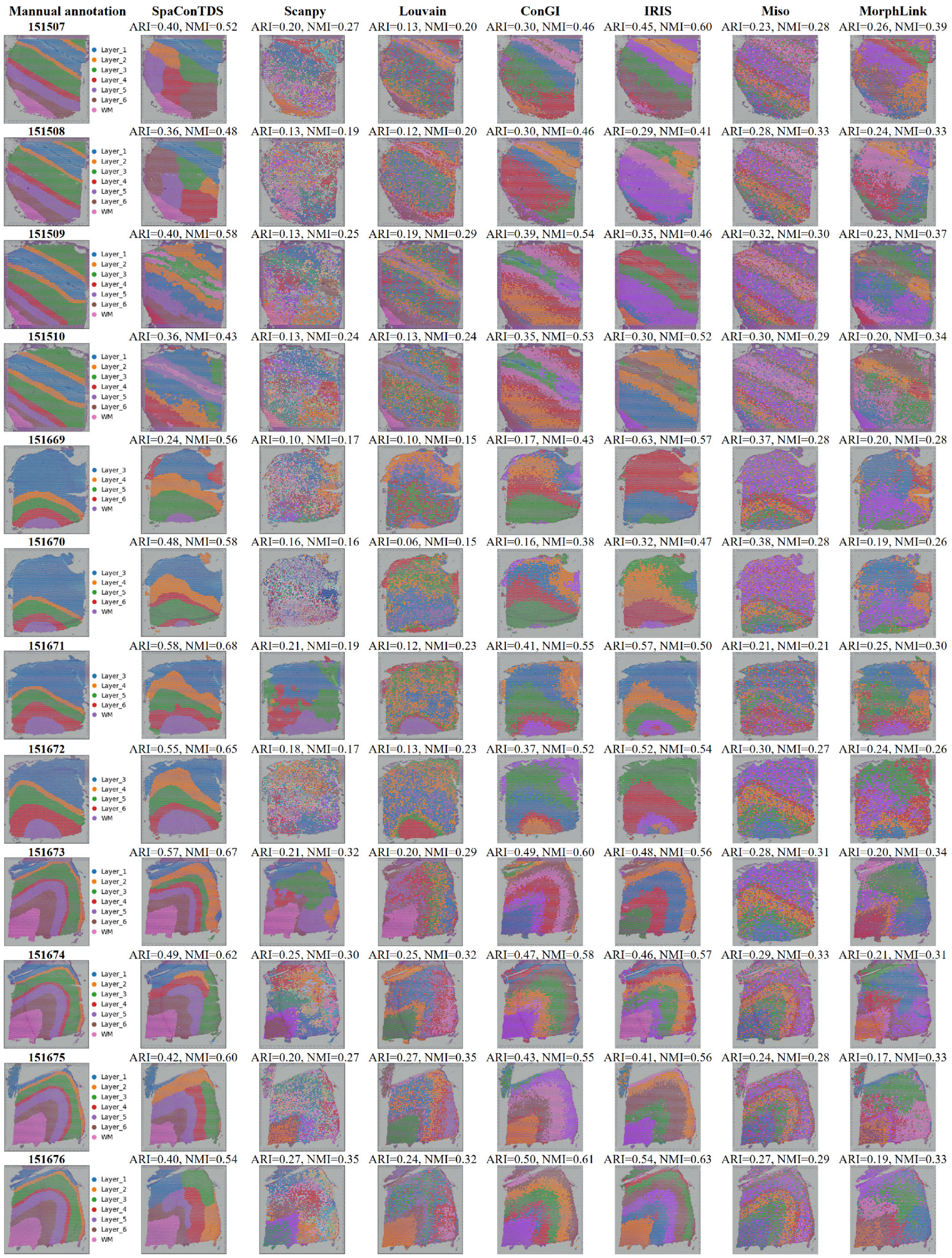

Fig S4. Manual annotations and comparison of spatial domains identified by SpaConTDS, scanpy, Louvain , ConGI, IRIS, Miso and MorphLink on the 12 slices of DLPFC dataset with ARI and NMI.
