# Supplementary material for: SpaConTDS: A multimodal contrastive learning framework for identifying spatial domains by applying tuple disturbing strategy
Source: PLoS Comput Biol. 2026 Jan 29;22(1):e1013893. doi: 10.1371/journal.pcbi.1013893 (PMC12854462; doi:10.1371/journal.pcbi.1013893)
Supplement: S5 Fig — (A) Manual annotations and comparison of spatial domains identified by SpaConTDS, STAGATE, ConST, GraphST, stLearn, SpaGCN, scanpy, Louvain, ConGI, IRIS, Miso, and MorphLink on IDC dataset with DB index. (B) Manual annotations and comparison of spatial domains identified by SpaConTDS, STAGATE, ConST, GraphST, stLearn, SpaGCN, scanpy, Louvain, ConGI, IRIS, Miso, and MorphLink on human breast cancer dataset with ARI and NMI. (C)Violin plots of expression of DEGs (SERHL2, CRISP3, DUSP23, EIF3H, UBE2S, IGFBP5, NUPR1, COX7C) in subcluster 2 and 15 versus other clusters. (D)Top: raw gene expression patterns of DEGs. Bottom: denoised gene expression patterns of DEGs. (PDF) [file pcbi.1013893.s007.pdf]

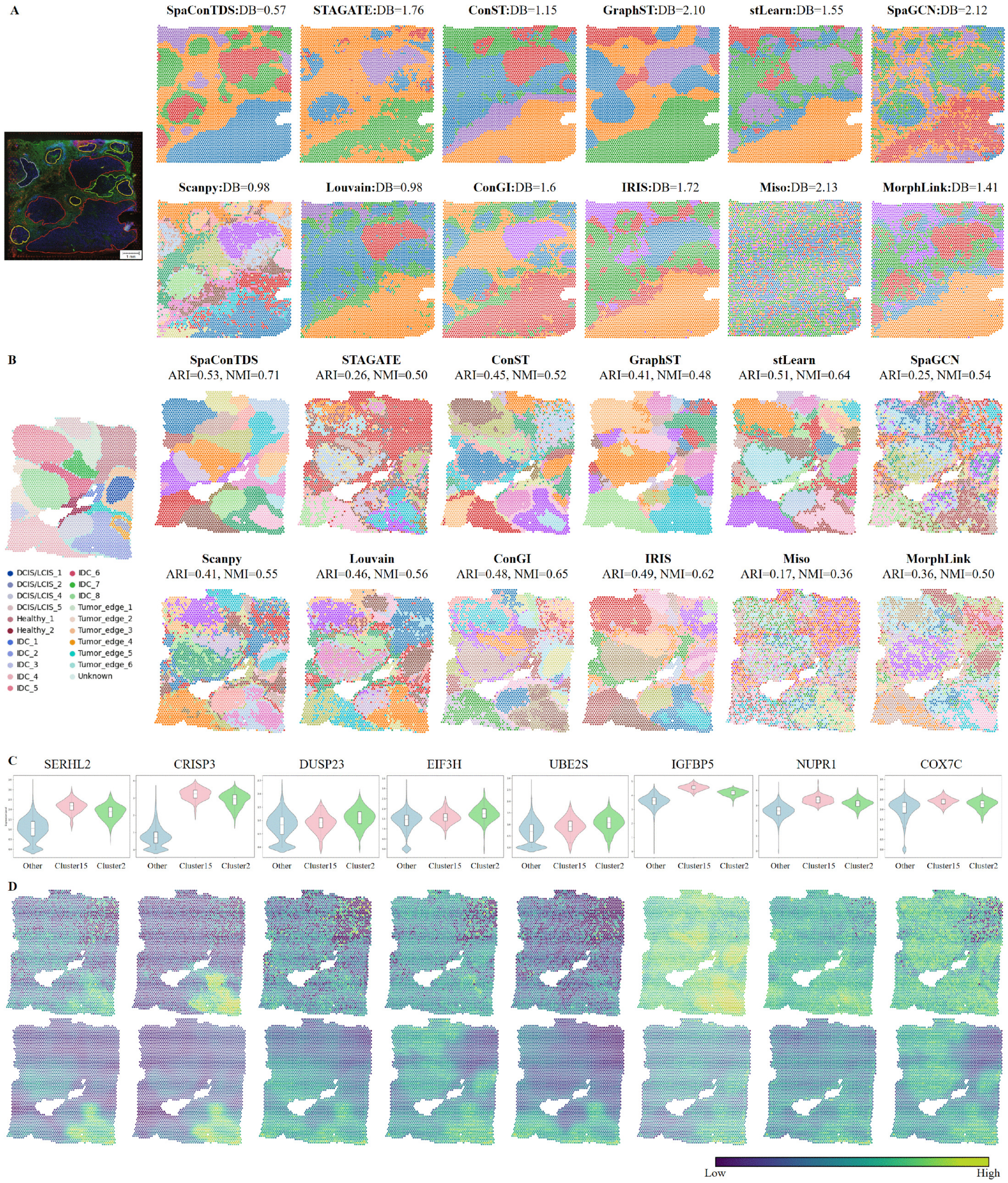

Fig S5. Visualization of differentially expressed genes (DEGs) supporting the detailed identified domains. (**A**) Manual annotations and comparison of spatial domains identified by SpaConTDS, STAGATE, ConST, GraphST, stLearn, SpaGCN, scanpy, Louvain , ConGI, IRIS, Miso and MorphLink on IDC dataset with DB index. (**B**) Manual annotations and comparison of spatial domains identified by SpaConTDS, STAGATE, ConST, GraphST, stLearn, SpaGCN, scanpy, Louvain , ConGI, IRIS, Miso and MorphLink on human breast cancer dataset with ARI and NMI. (**C**)Violin plots of expression of DEGs (SERHL2, CRISP3, DUSP23, EIF3H, UBE2S, IGFBP5, NUPR1, COX7C) in subcluster 2 and 15 versus other clusters. (**D**)Top: raw gene expression patterns of DEGs. Bottom: denoised gene expression patterns of DEGs.
