# Supplementary material for: SpaConTDS: A multimodal contrastive learning framework for identifying spatial domains by applying tuple disturbing strategy
Source: PLoS Comput Biol. 2026 Jan 29;22(1):e1013893. doi: 10.1371/journal.pcbi.1013893 (PMC12854462; doi:10.1371/journal.pcbi.1013893)
Supplement: S6 Fig — (A) UMAP plots after batch effect correction on 4 DLPFC slices. (B) UMAP plots after batch effect correction on human placental bed dataset slices. (C) Spatial domains identified by SpaConTDS on human placental bed dataset. (PDF) [file pcbi.1013893.s008.pdf]

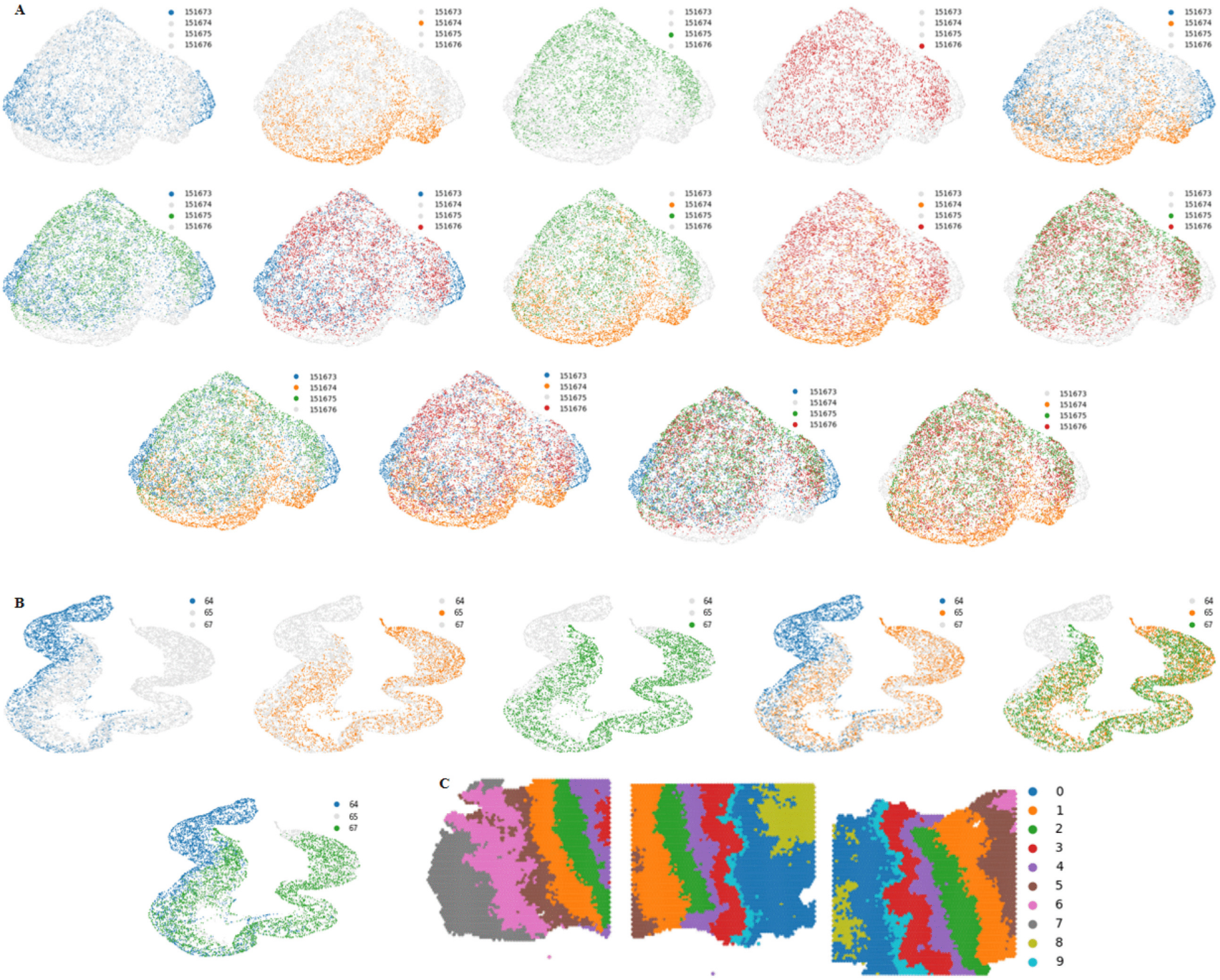

Fig S6. Batch effect correction ability of SpaConTDS. (**A**) UMAP plots after batch effect correction on 4 DLPFC slices. (**B**) UMAP plots after batch effect correction on human placental bed dataset slices. (**C**) Spatial domains identified by SpaConTDS on human placental bed dataset.
