# Supplementary material for: SpaConTDS: A multimodal contrastive learning framework for identifying spatial domains by applying tuple disturbing strategy
Source: PLoS Comput Biol. 2026 Jan 29;22(1):e1013893. doi: 10.1371/journal.pcbi.1013893 (PMC12854462; doi:10.1371/journal.pcbi.1013893)
Supplement: S7 Fig — (PDF) [file pcbi.1013893.s009.pdf]

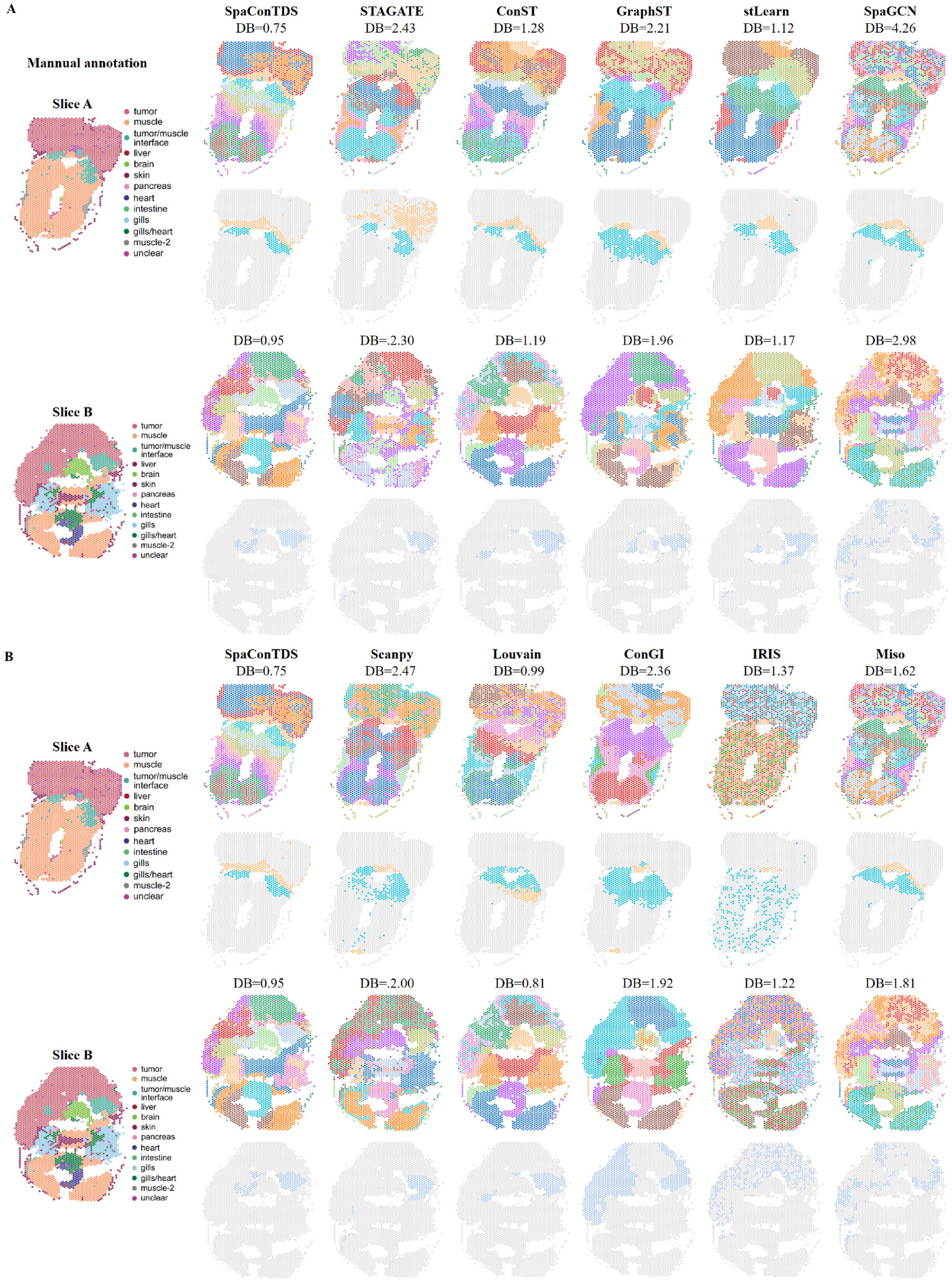

Fig S7. **Comparison of spatial clustering and interface domains identified by SpaConTDS, STAGATE, ConST, GraphST, stLearn, SpaGCN, scanpy, Louvain , ConGI, IRIS, Miso and MorphLink on slices A and B with DB index.**
