# Supplementary material for: SpaConTDS: A multimodal contrastive learning framework for identifying spatial domains by applying tuple disturbing strategy
Source: PLoS Comput Biol. 2026 Jan 29;22(1):e1013893. doi: 10.1371/journal.pcbi.1013893 (PMC12854462; doi:10.1371/journal.pcbi.1013893)
Supplement: S8 Fig — (PDF) [file pcbi.1013893.s010.pdf]

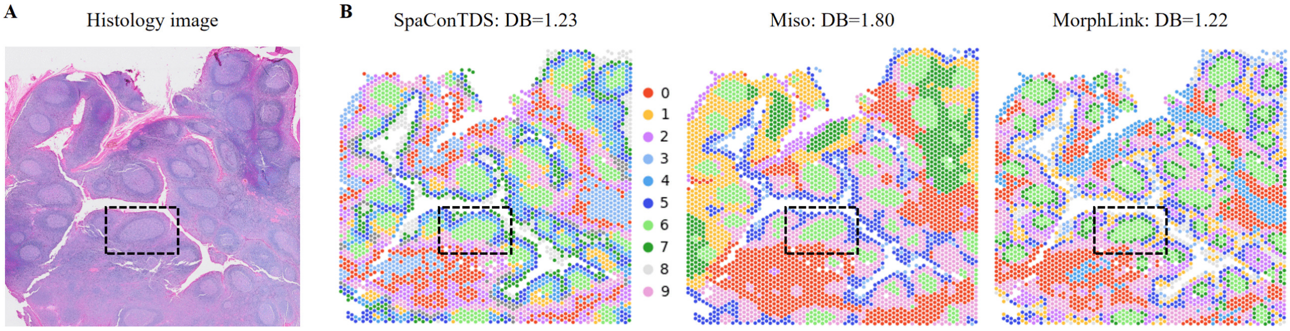

Fig S8. Comparison of spatial domains identified by SpaConTDS, Miso, and MorphLink on the human tonsil dataset comprising three modalities with DB index.
